# Unveiling the role of HP1α-HDAC1-STAT1 axis as a therapeutic target for HP1α-positive intrahepatic cholangiocarcinoma

**DOI:** 10.1186/s13046-024-03070-3

**Published:** 2024-05-30

**Authors:** Fei Xiong, Da Wang, Wei Xiong, Xin Wang, Wen-hua Huang, Guan-hua Wu, Wen-zheng Liu, Qi Wang, Jun-sheng Chen, Yi-yang Kuai, Bing Wang, Yong-jun Chen

**Affiliations:** 1 Department of Biliary‑Pancreatic Surgery, Tongji Hospital, Tongji Medical College, Huazhong University of Science and Technology, No.1095 Jiefang Road, Wuhan, Hubei 430074 China; 2 Department of General Surgery, Beijing Friendship Hospital, Capital Medical University Beijing, Beijing, 100050 China; 3 Department of Orthopedics, Tongji Hospital, Tongji Medical College, Huazhong University of Science and Technology, Wuhan, Hubei 430074 China; 4 Departement of Pediatric Surgery, Wuhan Children’s Hospital, Tongji Medical College, Huazhong University of Science and Technology, Hubei, 430016 China; 5 Department of Emergency, Tongji Hospital, Tongji Medical College, Huazhong University of Science and Technology, Wuhan, Hubei 430074 China

**Keywords:** Histone modification, HP1α, HDACi, STAT1, Interferon

## Abstract

**Background:**

Intrahepatic cholangiocarcinoma (ICCA) is a heterogeneous group of malignant tumors characterized by high recurrence rate and poor prognosis. Heterochromatin Protein 1α (HP1α) is one of the most important nonhistone chromosomal proteins involved in transcriptional silencing via heterochromatin formation and structural maintenance. The effect of HP1α on the progression of ICCA remained unclear.

**Methods:**

The effect on the proliferation of ICCA was detected by experiments in two cell lines and two ICCA mouse models. The interaction between HP1α and Histone Deacetylase 1 (HDAC1) was determined using Electrospray Ionization Mass Spectrometry (ESI-MS) and the binding mechanism was studied using immunoprecipitation assays (co-IP). The target gene was screened out by RNA sequencing (RNA-seq). The occupation of DNA binding proteins and histone modifications were predicted by bioinformatic methods and evaluated by Cleavage Under Targets and Tagmentation (CUT & Tag) and Chromatin immunoprecipitation (ChIP).

**Results:**

HP1α was upregulated in intrahepatic cholangiocarcinoma (ICCA) tissues and regulated the proliferation of ICCA cells by inhibiting the interferon pathway in a Signal Transducer and Activator of Transcription 1 (STAT1)-dependent manner. Mechanistically, STAT1 is transcriptionally regulated by the HP1α-HDAC1 complex directly and epigenetically via promoter binding and changes in different histone modifications, as validated by high-throughput sequencing. Broad-spectrum HDAC inhibitor (HDACi) activates the interferon pathway and inhibits the proliferation of ICCA cells by downregulating HP1α and targeting the heterodimer. Broad-spectrum HDACi plus interferon preparation regimen was found to improve the antiproliferative effects and delay ICCA development in vivo and in vitro, which took advantage of basal activation as well as direct activation of the interferon pathway. HP1α participates in mediating the cellular resistance to both agents.

**Conclusions:**

HP1α-HDAC1 complex influences interferon pathway activation by directly and epigenetically regulating STAT1 in transcriptional level. The broad-spectrum HDACi plus interferon preparation regimen inhibits ICCA development, providing feasible strategies for ICCA treatment. Targeting the HP1α-HDAC1-STAT1 axis is a possible strategy for treating ICCA, especially HP1α-positive cases.

**Supplementary Information:**

The online version contains supplementary material available at 10.1186/s13046-024-03070-3.

## Introduction

Based on the anatomical location, cholangiocarcinoma is divided into four types: intrahepatic, perihilar, and extrahepatic cholangiocarcinoma (ICCA, PCCA and ECCA) and gallbladder cancer (GBC). ICCA is a heterogeneous group of malignant tumors comprising approximately 20% of all cholangiocarcinoma and is characterized by a high degree of malignancy and atypical early symptoms. The incidence of ICCA is relatively high in certain endemic areas with a higher incidence of inflammatory biliary diseases, and the incidence rate is increasing yearly [1]. Owing to the late-stage presentation, resistance to comprehensive treatments, and extremely high rate of postsurgical recurrence, the 5-year overall survival rate of ICCA is lower than 10% [2]. Therefore, the identification of novel molecular targets is critical.

Heterochromatin Protein 1α (HP1α, encoded by the CBX5 gene) is one of the most important HP1 family members and is a non-histone chromosomal protein involved in transcriptional silencing via heterochromatin formation and structural maintenance [3]. Many studies have shown that the distribution of HP1α on polytene chromosomes is not restricted to the chromocenters or telomeres. HP1α binds to chromatin mainly through direct interactions with modified histones, especially trimethylated H3K9 (H3K9me3), through the Chromo domain, and by interacting with other proteins through the Chromo shadow domain [4]. In cancerous lesions, HP1α is involved in the regulation of malignant behaviors, such as cell proliferation and cell cycle progression. Downregulation of HP1α has been demonstrated to inhibit the malignant biological behaviors of lung cancer, cervical cancer, and prostate cancer cells, for example, by impairing proliferation and inducing apoptosis [5–7]. Previously, we found that downregulation of Dicer and CyclinD1, which are binding proteins of HP1α, inhibited the proliferation of ICCA cells [8, 9]. A better understanding of the exact mechanism of HP1α is required for the development of effective ICCA therapeutics.

Many cancerous lesions are characterized by suppressed intrinsic interferon (IFN) signaling and impaired immune responses [10]. IFN stimulation can occur in inflammatory diseases, but IFN signaling can inhibit the development of malignancies and is induced by multiple factors, such as, poly (ADP-ribose) polymerase inhibitors and IFN preparations (e.g., IFN-α2b) [11]. Three types of antiproliferative and anti-infection IFN signaling were observed: type I, II, and III (IFN-I, II, and III) signaling. Endogenous IFN-α and IFN-α variants mainly stimulate IFN-I signaling, although crosstalk between the three signaling pathways is often observed [10]. The Food and Drug Administration approved IFN-α2b as a treatment for hairy cell leukemia, renal cell cancer and melanoma. In a clinical trial of advanced ICCA, Kasai et al. found that ICCA patients could benefit from the IFN-α2b plus 5-fluorouracil regimen [12]. However, alternative regimens are needed, and a better understanding of the molecular mechanisms is needed to overcome potential resistance to immunotherapy.

In this study, we found that HP1α regulated cell proliferation in the context of ICCA. Downregulation of HP1α significantly stimulates interferon signaling by transcriptionally upregulating Signal Transducer and Activator of Transcription 1 (STAT1). The expression level of STAT1 can be regulated by the acetylation of histone H3 and the HP1α-Histone Deacetylase 1 (HDAC1) complex, necessitating the application of a regimen containing a histone deacetylase inhibitor (HDACi) plus IFN preparation, which was further validated in ICCA cell lines and mouse models. Compared with specific inhibitors, broad-spectrum HDACi has been shown to downregulate HP1α expression. Our study demonstrates the role of HP1α and IFN signaling as potential therapeutic targets in ICCA and provides an option for ICCA treatment.

## Materials and methods

### Bioinformatic analysis

Six expression microarrays (GSE4465, GSE18668, GSE32225, GSE32879, GSE76297 and GSE100705) and four genomic binding analysis datasets (GSE19465, GSE17312, GSE89212 and GSE89128) were downloaded from the Gene Expression Omnibus (GEO) database [13–20]. Detailed information is provided in Table S1. The expression dataset of CCA in The Cancer Genome Atlas (TCGA) was downloaded from the UCSC Xena database. In this study, the fold-change values were obtained by logarithmic (log2FC) transformation. False-positive results were avoided by calculating of adjusted P values (adj. P) values using the Benjamini-Hochberg procedure. The differentially expressed genes (DEGs) were defined with the following cutoff values: adj. *P* < 0.05 and |log2FC| > 0.8. The tools and databases used for the bioinformatics analysis are summarized in Table S2 [21–29].

## Cell culture

Human ICCA cell lines (HUCCT1, HCCC-9810, RBE, HUH28, and SSP-25) were maintained in our laboratory and cultured in RPMI-1640 medium. The kidney cell line, HEK-293T, was maintained in our laboratory and cultured in DMEM. All cell culture media were supplemented with 10% fetal bovine serum (FBS), 100 U/mL penicillin, and 100 µg/mL streptomycin at 37 °C in a humidified incubator with 5% CO2. The mycoplasma was detected by colorimetric method with Mycolor One-Step Mycoplasma Detector (Vazyme, China) according to the manufacturer’s instructions.

### Tissue microarrays, immunohistochemical (IHC) analysis and HE staining

The difference in HP1α expression between CCA and para-cancerous tissues was evaluated using a tissue microarray purchased from Outdo Biotech, China, containing 36 CCA samples and nine para-cancerous tissue samples. Additionally, 40 surgical ICCA and 40 paired para-cancerous tissues used for IHC and 90 surgical ICCA and 90 paired para-cancerous tissues used for real-time quantitative PCR (RT-qPCR) were collected from the Department of Biliary-Pancreatic Surgery, Tongji Hospital of Huazhong University of Science and Technology, Wuhan, China. The follow-up of these 90 ICCA patients was performed after surgery, and the date of death or last follow-up was recorded. All the research was conducted in accordance with both the Declarations of Helsinki and Istanbul. The experimental protocols were approved by the Ethics Committee of Tongji Hospital of Huazhong University of Science and Technology (#TJ-IRB20230927). All patients signed informed consent forms.

The tissue samples were fixed with 4% polyoxymethylene and subjected to embedding and sectioning at 5 μm thickness. For HE staining, the sections were sequentially stained with hematoxylin and eosin. For IHC analysis, samples were deparaffinized, rehydrated and subjected to antigen retrieval. Then, the samples were blocked with 5% bovine serum albumin (BSA) prior to overnight incubation with primary antibodies at 4 °C (listed in Table S3). The next day, tissue samples were incubated with horseradish peroxidase (HRP)-conjugated secondary antibodies prior to DAB and hematoxylin staining. The intensity of staining (0, negative; 1, weak; 2, moderate; and 3, strong) and the percentage of positive cells (0, 0%; 1, 1–25%; 2, 26-50%; 3, 51–75%; and 4, 76-100%) were evaluated in a blinded manner. Finally, the IHC score was calculated (IHC score = staining intensity × percentage of positive cells).

### Reagents and siRNA transfection

The inhibitors used in this study were as follows: Trichostatin A (TSA; HY-15144, 0.5 µM, 24 h), PF-06700841 (HY-112708, 50 nM, 24 h), decitabine (HY-A0004, 5 µM, 48 h), and GSK-J1 (HY-15648, 10 µM, 48 h) were purchased from MedChemExpress, China. Valproic acid (S1168, 1 µM, 48 h), Santacruzamate A (S7595, 20 µM, 48 h) and ACY-775 (S0864, 5 µM, 48 h) were purchased from Selleck, China. Human IFN-α2b (CYT-205, 60 ng/mL, 24 h) was purchased from Prospec (Israel). ICCA cells were transfected with siRNA (50 nM) and nonsense siRNA (50 nM) with Lipofectamine 2000 (Thermo Fisher Scientific, USA) following the manufacturer’s instructions. The siRNA constructs were synthesized by Sangon Biotech (China), and all the sequences are listed in Table S4.

### Plasmid construction and lentiviral transduction

Full-length and truncated sequences of Tripartite Motif Containing 28 (TRIM28), HDAC1 and CBX5, namely, full-length HDAC1 (Flag tagged, NM_004964.3), full-length CBX5 (HA tagged, NM_001127322.1), full-length TRIM28 (Flag tagged, NM_005762.3), truncated HDAC1 (lacking the histone deacetylase or disordered region) and truncated CBX5 (lacking the chromo domain or shadow domain), were generated by PCR and were inserted separately into the plasmid vector pHAGE-puro. Gene-specific small hairpin RNAs were synthesized by Sangon Biotech and were inserted into the pLKO.1-puro vector. HEK293T cells were transfected with the vectors mentioned above, along with the envelope plasmid pMD2.G and the packaging plasmid psPAX. The supernatant was filtered and collected after three days. ICCA cells were infected with the specific lentiviruses in the presence of HiTransG (1:25, GeneChem, China) for 24 h and then treated with 1 µg/ml puromycin to establish stable cell lines.

### RNA extraction, RT‒qPCR and RNA sequencing (RNA-seq)

Total RNA from cell and tissue samples was extracted with RNA Extraction Reagent (Vazyme) and reverse transcribed into cDNA using HiScript III RT SuperMix for qPCR (+ gDNA Wiper) (Vazyme) according to the manufacturer’s instructions. RT-qPCR was performed using Hieff® qPCR SYBR Green Master Mix (No ROX, Yeasen, China) and specific primers (Table S4) in an iQ5™ quantitative PCR system (Bio-Rad, USA). Expression levels were calculated using the 2 ^− ΔΔCt^ method. Primers were synthesized by Sangon Biotech.

Total RNA samples were used for RNA-seq performed by HaploX (China). Quality was evaluated using a 4200 TapeStation system (Agilent, USA) and quantified using a Life Invitrogen Qubit 3.0 fluorometer (Thermo Fisher Scientific). The samples were used for library preparation, followed by RNA-seq by Illumina PE150 sequencing (Illumina, USA). The raw data were used for further bioinformatics analysis.

### Western blot

Fresh cell samples were lysed with RIPA buffer in the presence of proteinase inhibitor cocktail and PhosSTOP phosphatase inhibitor (Roche, Switzerland). Total protein was extracted, mixed with 5× loading buffer, and denatured at 95 °C for 5 min. Proteins in individual lysate samples (30 µg per lane) were separated by sodium dodecyl sulfate‒polyacrylamide gel electrophoresis and transferred to nitrocellulose membranes (Millipore, USA). Then, the membranes were blocked with 5% BSA and incubated with primary antibodies overnight at 4 °C. The next day, the membranes were incubated with secondary antibodies, and the signals were visualized using enhanced chemiluminescence (Thermo Fisher Scientific). The results were analyzed using Image Lab software (Bio-Rad).

### Purification and isolation of nuclear and cytoplasmic proteins

Nuclear and cytoplasmic proteins were purified and isolated with Nuclear and Cytoplasmic Protein Extraction Kit (Beyotime, China). Briefly, ICCA cells were mixed with cytoplasmic extraction buffer A followed by vigorous vortexing and incubation in an ice bath. Cytoplasmic extraction buffer B was then added, followed by vigorous vortexing and centrifugation. The supernatant was collected as a cytoplasmic sample. The precipitate was mixed with nuclear extraction buffer, followed by vigorous vortexing, incubation in an ice bath, and centrifugation. The supernatant was collected as a nuclear sample for further analysis.

### Coimmunoprecipitation (co-IP) and Electrospray Ionization Mass Spectrometry (ESI-MS)

For the exogenous co-IP assay, HEK293T cells were transfected with expression vectors carrying full-length or truncated coding sequences. For the endogenous co-IP assay, wild-type ICCA cells were cultured without any specific treatment. The cells were harvested and lysed with IP buffer (20 mM Tris-HCl [pH 7.4], 150 mM NaCl, 1 mM EDTA, and 1% NP-40) in the presence of proteinase inhibitor cocktail and PhosSTOP. The samples were subjected to ultrasonication and centrifugation. 5% of the supernatant was collected as the input sample, and the remaining supernatant was incubated with primary antibodies and protein A + G agarose beads (Med Chem Express). The next day, the agarose beads were washed with IP buffer and heated to 95 °C in 2× loading buffer as the IP sample. All the protein samples were used for western blotting analysis. The IP samples of HEK293T cells transfected with the HP1α expression vectors and control vectors were used for ESI-MS analysis. The analysis was performed by Applied Protein Technology (China).

### Enzyme linked Immunosorbent Assay (ELISA)

The supernatants of ICCA cells were collected without dilution. The concentrations of IFN-α and IFN-γ were evaluated using a Human IFNA1 ELISA Kit (KE00044, Proteintech, China) and a Human IFN-gamma ELISA Kit (RK00015, ABclonal, China) according to the manufacturer’s instructions. The supernatant and the reaction mixture were incubated at 37 °C, and the absorbance at 450 nm was measured using a MULTISKAN FC microplate reader (Bio-Rad).

### Cleavage under targets and tagmentation (CUT & tag)

A Hyperactive Universal CUT&Tag Assay Kit for Illumina (Vazyme) was used to evaluate the genomic occupancy of DNA binding proteins. Briefly, 1 × 10^5^ ICCA cells were collected and the nuclei were extracted. Specific DNA fragments were obtained as previously described [30]. The DNA library was constructed using a TruePrep Index Kit V2 (Vazyme) according to the manufacturer’s instructions. The library was sequenced on the Illumina HiSeq 2000 platform (Illumina) by HaploX.

### Luciferase reporter assay

The vector carrying the sequence 1000 bp upstream of the transcription start site (TSS) and the corresponding control vector (Promoter-STAT1 and Promoter-NC, respectively) were constructed by GeneChem. HEK293T cells were plated in 24-well plates and treated with inhibitors (TSA or DMSO) or transfected with these vectors along with HP1α expression vectors or normal control vectors. The Renilla luciferase vector was also transfected as Renilla luciferase control. After 48 h, the cell lysates were harvested, and luciferase activity was measured using a Dual Luciferase Reporter Assay Kit (Vazyme) and a Dual Luciferase Assay system (Promega, USA). The results were normalized to the Renilla luciferase activity.

### Chromatin immunoprecipitation (ChIP)

ChIP was performed as previously described [9]. Briefly, ICCA cells were collected, fixed with 1% formaldehyde and then neutralized with 10% glycine. After ultrasonication, the DNA-protein complexes were precipitated using primary antibodies and protein A + G agarose beads. The abundance of precipitated DNA was measured using ChIP-qPCR and normalized to that of the IgG control group. Primers are listed in Table S4.

### Flow cytometry

The percentage of apoptotic cells and cell cycle distribution were evaluated using an Annexin V-FITC/PI apoptosis kit (70-AP101-100, MultiSciences, China) and a cell cycle staining kit (CCS012, MultiSciences), respectively. ICCA cells (2 × 10^6^ per sample) were harvested, stained with Annexin V-FITC and PI, and analyzed by flow cytometry using a Becton-Dickinson FACScan System (BD Biosciences, USA). The data were analyzed using the system software.

### Proliferation assay

A CCK-8 assay was used to evaluate overall ICCA cell proliferation. For the evaluation of gene function, 0.8 × 10^3^ cells per well were plated in 96-well plates and cultured for 0, 24, 48, 72, or 96 h. To evaluate of inhibitory effects, 3 × 10^3^ ICCA cells were plated in each well and cultured for 0, 24, 48, or 72 h. CCK-8 solution (Boster, China) was used, and the absorbance at 450 nm was measured using a MULTISKAN FC microplate reader.

Clonality was assessed using a colony formation assay. ICCA cells (1 × 10^3^) were plated in 6-well plates and cultured for 2 weeks. Colonies were fixed with 4% polyoxymethylene and stained with crystal violet. The colonies were then counted.

### Wound healing assay

The invasive ability of ICCA cells was evaluated using a wound healing assay. ICCA cells were plated in 6-well plates and cultured until they reached confluence. A 200 µL pipette tip was used to generate linear scratches, and the cells were incubated with serum-free RPMI-1640 medium. Images were acquired 0 h and 24 h after wounding. The cell migration rate was used to evaluate the migration ability of ICCA cells [cell migration rate = (wound area at 0 h − wound area at 24 h)/wound area at 0 h].

### Mouse model

The animal experiments were approved by the Animal Experimentation Ethics Committee of Tongji Medical College (#TJH-202208006). Animal care and experimental procedures were performed according to the criteria outlined in NIH guidelines. The maximal tumor size permitted by their ethics committee was 1000 mm^3^. All the tumor lesions in this study were below 1000 mm^3^. All the animals were housed in a specific pathogen-free environment, including bedding, caging systems, and diet.

Conventional Cbx5 knockout mice model (C57BL/6NCbx5em1Cya) was constructed using CRISPR/Cas9-mediated genome engineering by Cyagen (China). Exons 3–5 of the Cbx5 gene were selected as target sites. No other known genes have been identified in the knockout region. Mice were genotyped by PCR, followed by sequence analysis, and divided into three groups (Cbx5+/+, Cbx5+/-, and Cbx5-/-). In this study, seven Cbx5+/+, seven Cbx5+/-, and five Cbx5-/- mice from the same generation were used, and all the mice were 5 weeks old. No special treatment was administered. The mice were euthanized and the lungs and livers were collected for further analysis.

C57BL/6 N mice were purchased from GemPharmatech (China) and ICCA tumors were induced by hydrodynamic tail vein injection. Thirty male C57BL/6J mice (5 weeks old) were randomly divided into six groups. The full-length coding sequence of the murine Cbx5 transcript NM_001110216.1 (encoding murine Hp1α protein) was inserted into the pT3-EF1α vector (Hp1α-OE) and the corresponding control vector was named Hp1α-NC. For the injection of each mouse, 15 µg of pT3-EF1α-myr-AKT1, 15 µg of pT3-EF1α-NICD, 3 µg of pCMV-SB, and 15 µg of Hp1α-OE or Hp1α-NC were diluted in 2 ml of saline and quickly injected over 5–7 s. Plasmids were obtained from Addgene (USA). All mice developed ICCA tumors. We failed to obtain murine IFN-α2b; therefore, IFN-α2 was used. After 1 week, the mice were administered TSA [Standard dose 0.5 mg/kg, intraperitoneal injection, diluted in 200 µL of the solvent recommended by the supplier (10% DMSO + 40% PEG300 + 5% Tween 80 + 45% saline)] and murine IFN-α2 (RP01725, ABclonal, 3 µg/kg according to the specific activity conversion, subcutaneous injection, diluted in 200 µL of double-distilled water with 1% FBS). Until the fifth week, all mice were euthanized, and ascites, kidneys, and livers were collected.

Fifteen female nude mice were purchased from GemPharmatech and were equally and randomly divided into three groups as previously described [30]. Briefly, HUCCT1 cells (2 × 10^6^/mouse) were injected subcutaneously into the upper right flank. All the mice developed subcutaneous tumors. The treatment plan was the same as that described above. After the mice were euthanized, tumors were collected.

### Statistical analysis

All data are presented as representative images of each group or as mean ± standard error of the mean from three separate experiments. Student’s t-test was used to compare the values between two subgroups, while ANOVA was used to analyze the differences among multiple groups using GraphPad Prism 8 (GraphPad, USA). Pearson correlation coefficient (r) was used to evaluate the correlation between the expression of the two genes. A significant correlation was defined as |r| > 0.35 and *P* < 0.05. Kaplan‒Meier analysis with log-rank test was used to evaluate survival differences. All statistical tests were two-sided, and statistical significance was set at *P* < 0.05.

## Results

### HP1α is upregulated in ICCA and regulates cell proliferation

**Fig. 1:**
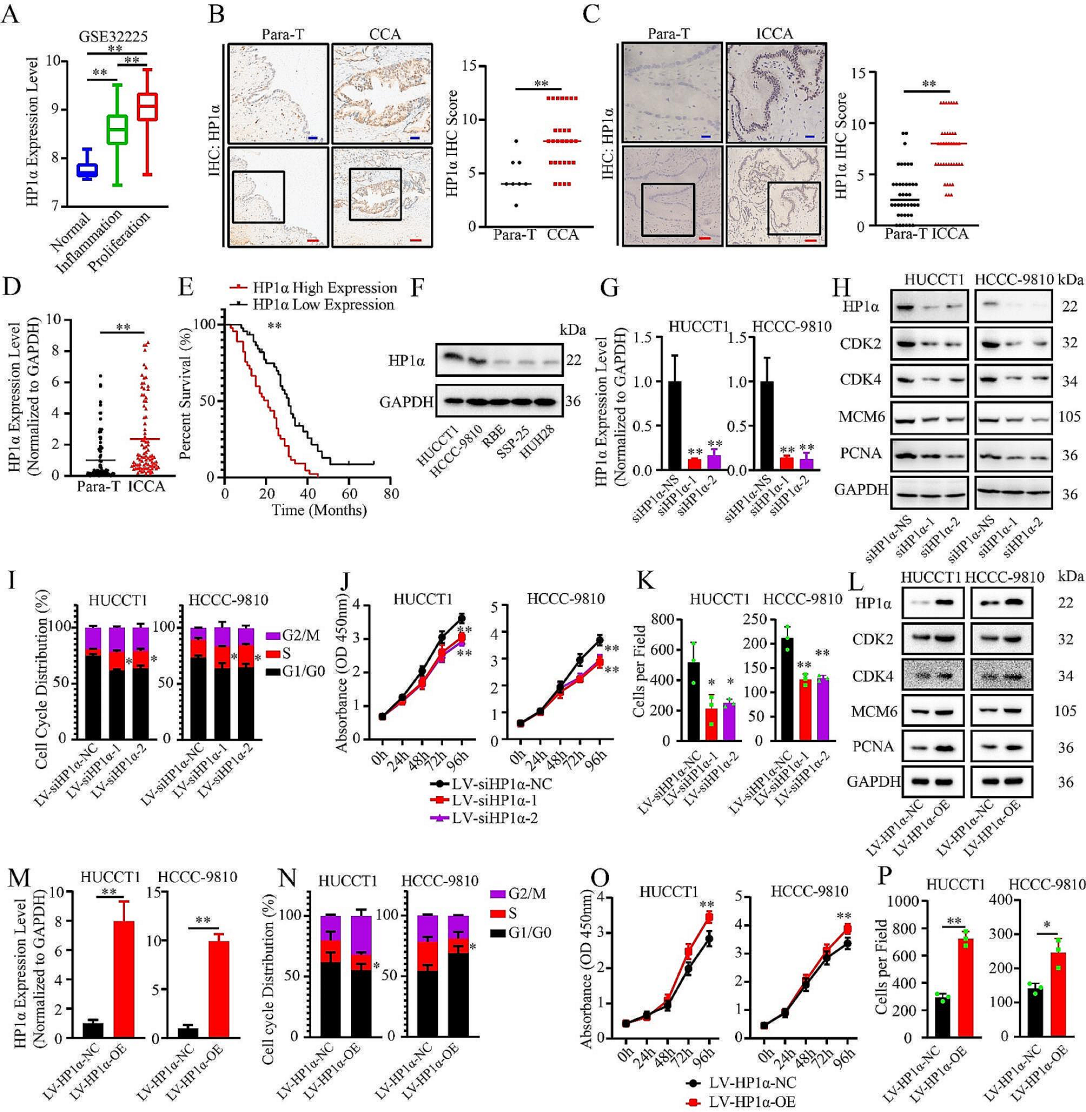
HP1α downregulation impairs the proliferation of ICCA cells. **A.** The difference in HP1α expression among different subgroup in GSE32225. Subgroups were divided according to the pathological features. Inflammation group contained the samples characterized by the hyperactivation of inflammation pathways. Proliferation groups contained the samples featured by the overaction of oncogenic pathways and lack of inflammation response. **B.** IHC result of the tissue microarray of CCA using HP1α antibodies (28 CCA samples and 8 normal samples). **C.** IHC result of the ICCA tissue samples using HP1α antibodies (40 ICCA samples and 40 normal samples). **D.** The difference in HP1α expression between ICCA tissue and para-cancerous tissue evaluated by RT-qPCR (90 ICCA samples and 90 normal samples). **E.** Survival analysis of ICCA cohort. The median of HP1α expression level was selected as the cutoff value. **F.** The difference in HP1α expression among five ICCA cell lines. **G.** RT-qPCR analysis of the effect of HP1α knockdown. **H.** Western blot analysis of HP1α and proliferation and cell cycle-promoting factors. **I.** Evaluation of the impact on the distribution of cell cycle upon HP1α knockdown. **J, K.** Results of the proliferation assay upon HP1α knockdown. **J.** CCK-8 assay. **K.** Colony formation assays. **L.** Western blot analysis of HP1α expression, proliferation, and cell cycle-promoting factors upon HP1α overexpression. **M.** RT-qPCR analysis of the effect of HP1α overexpression. **N.** Evaluation of the impact on the distribution of cell cycle upon HP1α overexpression. **O, P.** Results of the proliferation assay following HP1α overexpression. **O.** CCK-8 assay. **P.** Colony formation assay. Blue bar, 20 μm. Red bar, 50 μm. Para-T: para-cancerous tissue. *P < 0.05; **P < 0.01

To determine the role of HP1α in ICCA, we compared the expression of HP1α mRNA in four datasets that contained CCA and ICCA samples. Compared with the surrounding normal bile duct tissue, the mRNA of HP1α was upregulated in both CCA and ICCA tissues (Fig. 1A, S1A). This conclusion was validated by IHC and RT-qPCR of the tissue microarray and the two cohorts that we enrolled (Fig. 1B-D). High HP1α expression indicated poor prognosis in patients with ICCA, indicating the role of HP1α as a motivator of ICCA development (Fig. 1E). To explore the function of HP1α, we analyzed the genes whose expression was correlated with that of HP1α in GSE32225 with the most cancerous samples (149 ICCA samples) (Figure S1B). We found 2544 negatively correlated genes and 3573 positively correlated genes. Some of the correlated genes were involved in cell cycle regulation and pathogen infection (Figure S1C). Theoretical prediction showed that these genes were regulated by crucial transcription factors in the IFN pathway, including interferon regulatory factors (IRFs) and STATs (Figure S1D).

All the cell lines used in this study was free of mycoplasma (Figure S1E). HUCCT1 and HCCC-9810 were selected for further analysis because of their high HP1α expression levels (Fig. 1F). To validate the role of HP1α, we used specific siRNAs and knockdown lentiviral vectors to target the same sequences (Fig. 1G and H). We found that the protein levels of proliferation and cell cycle-promoting factors were downregulated upon HP1α knockdown (Fig. 1H). HP1α downregulation led to S-phase arrest in both the cell lines (Fig. 1I). Cell proliferation assays revealed that cell viability was significantly impaired (Fig. 1J K, and S1F). An HP1α expression vector was also constructed (Fig. 1L M). The aforementioned protein factors were upregulated upon HP1α upregulation (Fig. 1L). The distribution of S-phase cells decreased upon HP1α overexpression (Fig. 1N). The proliferation of both the cell lines was slightly enhanced (Fig. 1O and P, and S1G). For HP1α-negative cells, e.g., RBE, HP1α overexpression promoted the proliferation of tumor cells and decreased the ratio of tumor cells in S-phase (Figure S1H-S1K). However, apoptosis and invasion of ICCA cells were not significantly affected by HP1α knockdown (Figure S1L and S1M). Therefore, regulation of cell proliferation and cell cycle progression was identified as a key function of HP1α in ICCA. This conclusion was further validated by clinicopathological analysis, as we found that tumor lesion size was correlated with HP1α expression (Table 1).

**Table 1:** Association between HP1α expression and clinicopathological characteristics

Variable	Case (Number, %)	HP1α Expression Level		Significance
		High Expression (N=45)	Low Expression (N=45)	
Age (Year)	<=55	14	14	ns
	>55	31	31	
Gender	Female	20	16	ns
	Male	25	29	
Lesion Size (cm)	<=2	10	34	**
	>2	35	11	
T Classification	T1+T2	35	36	ns
	T3+T4	10	9	
N Classification	N0	36	32	ns
	N1	9	13	
Stage	I+II	29	24	ns
	III+IV	16	21	

**P<0.01. ns, not significant

### Downregulation of HP1α enhances endogenous basal IFN signaling

**Fig. 2:**
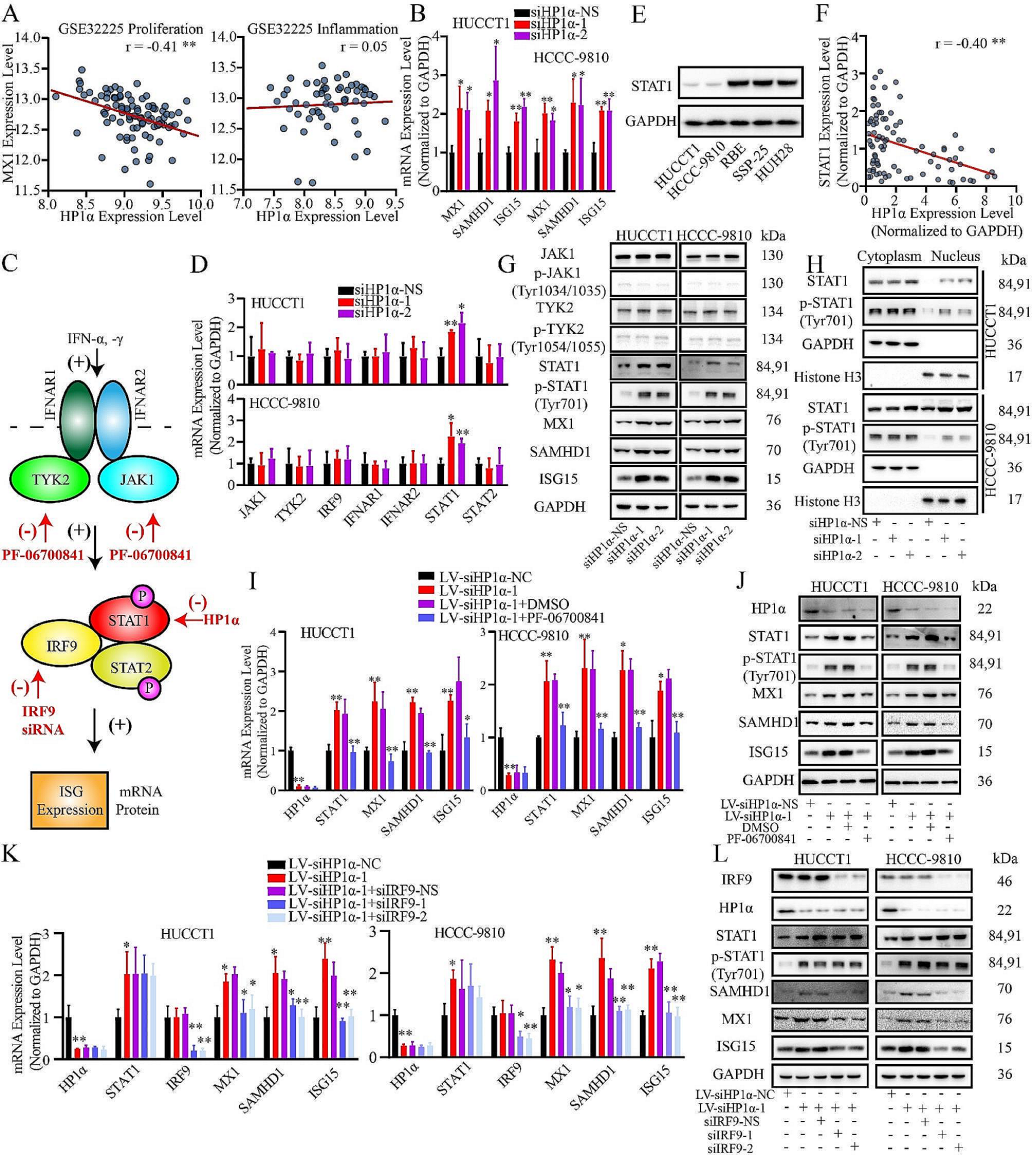
Downregulation of HP1α activates IFN signaling pathway. **A.** The correlation between the expression of MX1 and HP1α in the dataset of two distinct groups in GSE32225. **B.** RT-qPCR analysis of the expression of MX1, ISG15 and SAMHD1. **C.** Graphic illustration of the interventions mentioned in this section. PF-06700841 inhibited the IFN signaling by directly impairing the activation of JAK1 and TYK2. IRF9 could be knocked down by specific siRNAs. The activation level of IFN pathway could be indicated by the expression level of ISGs. **D.** RT-qPCR analysis of the expression of the upstream participants of IFN-I signaling **E.** The difference in STAT1 expression among five ICCA cell lines. **F.** Analysis of the correlation between the expression of HP1α and STAT1 in ICCA tissue (*N* = 90). **G.** Western blot analysis of the expression of the upstream participates of IFN-I signaling and the activation level of upstream kinases. **H.** Western blot analysis of the expression of STAT1 and p-STAT1 in cytoplasm and nucleus. **I, J.** Evaluation of the effect of inhibiting upstream kinases on the HP1α-STAT1 axis. **I.** RT-qPCR. **J.** Western blot. **K, L.** Effect of ISGF3 formation inhibition on the HP1α-STAT1 axis. **K.** RT-qPCR. **L.** Western blot. *P < 0.05; **P < 0.01

HP1α is recognized for its role as a transcriptional repressor [3]. In the GSE32225 dataset, ICCA cases were divided into inflammation and proliferation subgroups. The expression level of HP1α in the inflammation subgroup was lower than that in the proliferation subgroup, which was characterized by the activation of oncogenic pathways and poor prognosis rather than overactivation of inflammation pathways (Fig. 1A). We found that the expression of MX dynamin-like GTPase 1 (MX1), an IFN-stimulated gene (ISG) that is induced by IFN-α and involved in antitumor and antiviral responses [10], was negatively correlated with HP1α expression (Figure S1B). This correlation was only observed in the proliferation subgroup (Fig. 2A). Surprisingly, MX1 and another two known ISGs, SAM domain, HD domain 1 (SAMHD1), and ISG15, were upregulated following HP1α downregulation (Fig. 2B). To explore the relationship between HP1α and the IFN pathway, we measured the mRNA levels of upstream participants in the IFN-I pathway and found that only STAT1 was significantly upregulated upon HP1α knockdown (Fig. 2C and D). Among the ICCA cell lines, the expression of STAT1 was lower in HUCCT1 and HCCC-9810 cells, both of which met the criteria of the proliferation subgroup mentioned in GSE32225 and were characterized as HP1α-positive (Fig. 2E). The negative correlation between HP1α and STAT1 expression was validated in ICCA tissue samples (Fig. 2F). The protein levels of MX1, SAMHD1, ISG15, phosphorylated STAT1 (p-STAT1), and STAT1 were also elevated, whereas the activation levels of the upstream kinases Janus kinase 1 (JAK1) and tyrosine kinase 2 (TYK2), which function in IFN signal transduction, were not affected (Fig. 2G). In HP1α-negative cells, e.g., RBE, STAT1, p-STAT1 and ISGs were downregulated upon HP1α overexpression (Figure S1H). Additionally, STAT1 and p-STAT1 upregulation seemed to be significant in the nucleus, but not in the cytoplasm, a significant step for the activation of IFN signaling pathway (Fig. 2H).

To uncover the molecular mechanisms, we performed an ELISA and found that IFN proteins (e.g., IFN-α and IFN-γ) were barely detectable in the culture medium of either cell line (Figure S1N). Since no exogenous IFN was applied, we hypothesized that the activation of the IFN pathway might arise from endogenous mechanisms. We also examined the mRNA levels of the IL6-STAT3 pathway (recognized for inhibiting the IFN pathway), cGAS-STING pathway (mediating the secretion of IFN), and NF-κB pathway (regulating the expression of IRFs) components, but no significant differences were observed upon HP1α knockdown (Figure S1O-S1Q) [31, 32]. To validate the central node role of STAT1, we applied a specific inhibitor of JAK1 and TYK2, PF-06700841, and found that the induction of p-STAT1, STAT1, and ISG expression by HP1α knockdown was abrogated at the mRNA and protein levels (Fig. 2C and I, and 2J), indicating that the basal activation of IFN signaling was maintained and regulated the HP1α-STAT1 axis. After IRF9, an important component of downstream IFN-stimulated gene factor 3 (ISGF3), was knocked down, only the upregulation of ISGs induced by HP1α knockdown was abrogated (Fig. 2C and K, and 2L). Combined with the observation that the HP1α expression level was not influenced (Fig. 2I and L), these findings indicate that STAT1 is the central node in the interaction between HP1α and the IFN pathway.

The HP1α-STAT1 axis was further validated in conventional Cbx5 knockout mice. No significant atypia was observed in the liver or lung tissues, confirming the consistency of the tissue types for further analysis (Figure S1R). Using IHC and Western blotting, we found that Stat1 was upregulated by Cbx5 knockout in normal lungs, but not in normal livers (Figure S1S-S1U). Mx1 was not detected in either tissue, whereas Isg15 and Samhd1 were upregulated upon Cbx5 knockout in lung tissues. Notably, the induction of STAT1 and ISGs is still limited, especially when compared with the effects of cytokines [33]. Thus, the HP1α-STAT1 axis can only influence the basal level of IFN signaling.

### Downregulation of HP1α inhibits cell proliferation by upregulating STAT1 and activating IFN signaling

**Fig. 3:**
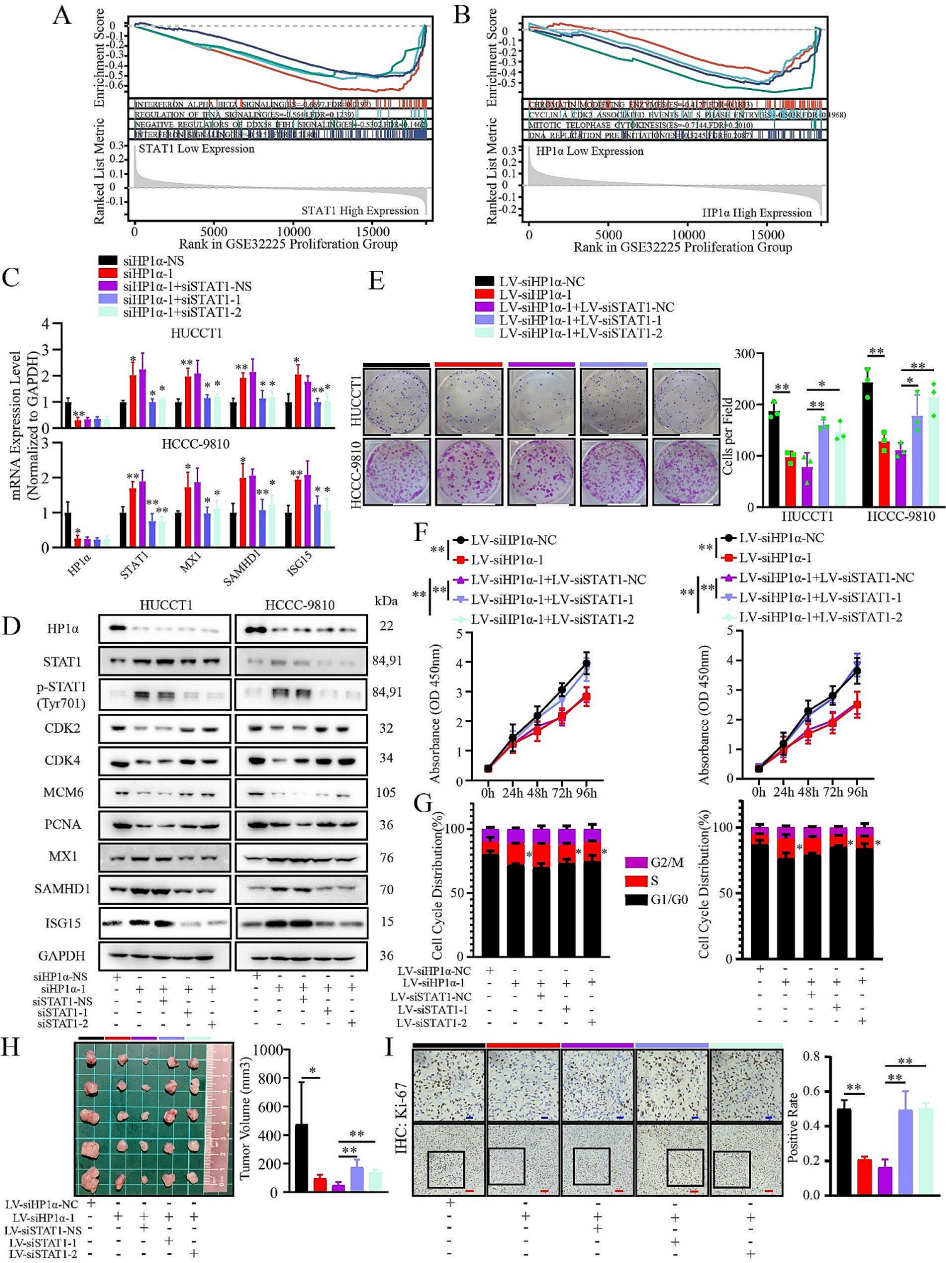
HP1α knockdown inhibits the proliferation of ICCA cells by upregulating STAT1 and IFN signaling induction. **A, B.** The results of GSEA analysis revealed the critical function of two genes based on the Reactome database with the dataset of the GSE32225 proliferation group. **(A)** STAT1. **(B)** HP1α. **C.** Evaluation of HP1α and STAT1 expression and IFN signaling activation by RT-qPCR upon STAT1 knockdown. **D.** Western blot analysis of HP1α and IFN signaling participants and proliferation-and cell cycle-promoting factors upon STAT1 knockdown. **E, F.** Results of the proliferation assay after STAT1 knockdown. **E.** Colony formation assay. White bar, 1.96 cm. **F.** CCK-8 assay. **G.** The distribution of the cell cycle of ICCA cells upon STAT1 knockdown. **H.** Presentation of the subcutaneous tumors of different groups. **I.** Evaluation of the Ki-67 positivity rate of subcutaneous tumors. **Blue bar**, 20 μm. **Red bar**, 50 μm. *P < 0.05; **P < 0.01

GSEA results showed that HP1α and STAT1 influenced cell proliferation and IFN pathway, respectively (Fig. 3A and B). To explore whether HP1α downregulation inhibits the proliferation of ICCA cells via STAT1 upregulation, we transduced specific STAT1-knockdown lentiviral vectors into HP1α-knockdown ICCA cells. The increase in the expression levels of p-STAT1 and the three ISGs was significantly mitigated, whereas HP1α expression was not affected (Fig. 3C and D). When HP1α-knockdown ICCA cells were transduced with the knockdown lentiviral vectors for the three ISGs, the inhibition of proliferation mediated by HP1α downregulation was abrogated, indicating the antiproliferative role of these ISGs (Figure S2A-S2C). Downregulation of proliferation and cell cycle factors was partly reversed by STAT1 knockdown (Fig. 3D), which was consistent with the proliferation assay results (Fig. 3E and F). The S-phase arrest induced by HP1α knockdown was mitigated by STAT1 knockdown (Fig. 3G). This conclusion was further validated in nude mouse models, as STAT1 knockdown significantly reversed the decrease in the volume and Ki-67-positive rate of HUCCT1 xenograft tumors upon HP1α downregulation (Fig. 3H and I). Therefore, HP1α downregulation inhibits proliferation and cell cycle progression by transcriptionally upregulating STAT1 and ISG expression and activating IFN signaling.

### STAT1 is regulated by HDAC1 and histone acetylation

**Fig. 4:**
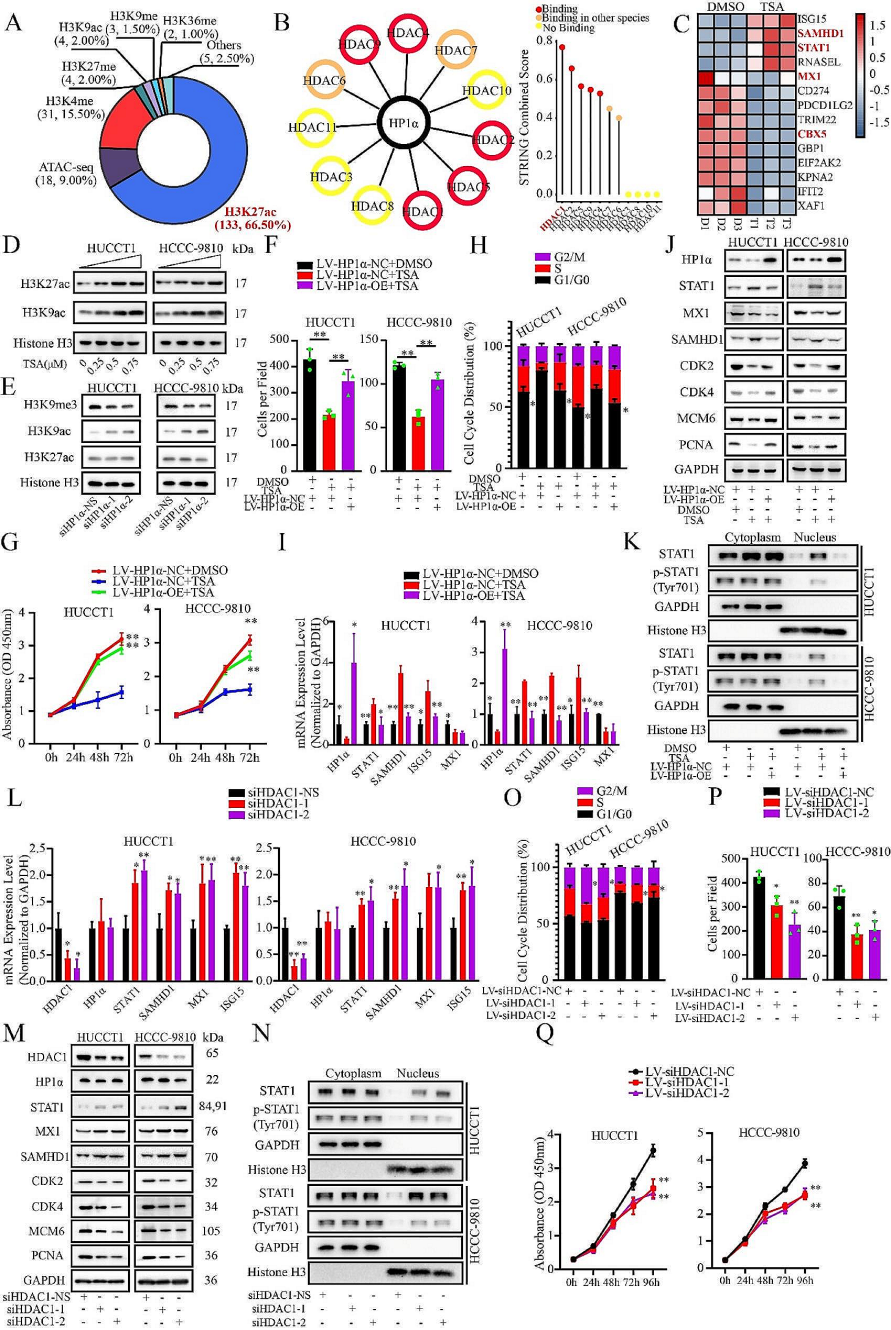
STAT1 is regulated by HDAC1 and histone acetylation. **A.** Summary of the histone marks located in the promoter region of STAT1 gene. **B.** Summary of the interaction between HDACs and HP1α. The combined score calculated by STRING was shown. HDACs were divided into three groups according to whether the interaction was previous found in human tissues. **C.** The heatmap of the RNA-seq data of TSA-treated ICCA cells. The gradual change from red to blue represents changes in gene expression from high to low. **D.** Western blot analysis of the total histone acetylation induced by TSA treatment. **E.** Western blot analysis of the total level of selected histone marks upon HP1α knockdown. **F, G.** Results of the proliferation assay upon TSA treatment and HP1α overexpression. **F.** Colony formation assay. **G.** CCK-8 assay. **H.** Analysis of the cell cycle distribution in ICCA cells upon TSA treatment and HP1α overexpression. **I.** RT-qPCR analysis of HP1α, STAT1, and ISGs expression upon TSA treatment and HP1α overexpression. **J.** Western blot analysis of HP1α and IFN signaling participants and proliferation and cell cycle-promoting factors after TSA treatment and HP1α overexpression. **K.** Western blot Analysis of the expression levels of intranuclear STAT1 and p-STAT1 upon TSA treatment and HP1α overexpression. **L, M.** Impact of HDAC1 knockdown on the expression of HP1α and IFN signaling **L.** RT-qPCR. **M.** Western blot. **O-Q.** Effect of HDAC1 knockdown on ICCA cell proliferation **O.** Cell cycle analysis. **P.** Colony formation assay. **Q.** CCK-8 assay. *P < 0.05. **P < 0.01

To determine the mechanism underlying the transcriptional regulation of the STAT1 gene, we searched online database and found that within 1000 bp upstream of the TSS region, 66.50% of the high-ranking terms were associated with acetylated H3K27 (H3K27ac) which could be regulated by HDAC (Fig. 4A, Table S5). The interaction between HP1α and HDACs was investigated (Fig. 4B). Binding sites in the STAT1 promoter region were predicted (Table S6). Three histone deacetylases (HDACs) were found to bind to both the HP1α protein and STAT1 promoter regions, i.e., HDAC1, HDAC2 and HDAC6. Additionally, we found that STAT1 expression could not be induced by targeting epigenetic modifications such as DNA methylation and H3K27me3 (Figure S2D, S2E).

To explore the relationship between STAT1 and HDACs, TSA was applied to HUCCT1 cells, and RNA-seq was performed (Fig. 4C). We found 3407 upregulated differentially expressed genes (DEGs) and 2717 downregulated DEGs, some of which were involved in the cell cycle, viral infection, and IFN pathway (Figure S2F). TSA significantly elevated the total levels of histone acetylation markers (Fig. 4A) in a dose-dependent manner (Fig. 4D). When HP1α was knocked down, the total H3K9me3 level decreased, whereas that of H3K9ac increased. The H3K27ac level seemed to be more stable (Fig. 4E), indicating that the local distribution should be the focus.

TSA, a known antiproliferative agent and broad-spectrum HDACi [34], was used for the proliferation assay of ICCA cells. The proliferation of both cell lines was significantly impaired upon TSA application, and G1/G0 arrest was observed. Both of these effects were partly rescued by HP1α overexpression (Fig. 4F and H, S2G). The expression levels of STAT1, p-STAT1, ISG15, and SAMHD1 increased, and the proliferation and cell cycle factors were downregulated (Fig. 4I and J). Synchronous elevation of intranuclear STAT1 and p-STAT1 levels was observed (Fig. 4K). Surprisingly, TSA decreased HP1α levels, indicating that TSA may be an upstream regulator of the HP1α-STAT1 axis. As HDACs are regarded as transcription inhibitors, HP1α downregulation cannot be explained by direct inhibition of HDACs. MX1 was not induced by TSA, a phenomenon that remains to be studied (Fig. 4I and J). The non-specific pharmacological action of broad-spectrum HDACi might be the reason, but this effect could be inferred to be antineoplastic and worthy of further study. Additionally, a relationship between TSA and the regulatory axis was found in other tissues (Figure S2H).

To determine which HDAC should be selected, ICCA cells were transfected with specific siRNAs targeting HDAC1, HDAC2, and HDAC6, none of which could be regulated by HP1α (Figure S2I). We found that only knockdown of HDAC1 slightly induced STAT1 and ISGs expression (Fig. 4L and M, S2J, S2K), which was further validated by specific inhibitors targeting these HDACs (Figure S2L). Compared with broad-spectrum HDACi, specific HDACi did not downregulate HP1α expression. Only specific HDAC1 inhibitor could slightly inhibit the proliferation of ICCA cells (Figure S2M). Similarly, HDAC1 knockdown led to cell cycle arrest in the G2/M phase in HUCCT1 cells and in the S phase in HCCC-9810 cells (Fig. 4O). HDAC1 knockdown impaired the proliferative ability of ICCA cells (Fig. 4P and Q, and S2N). Therefore, as a tumor-promoting factor, HDAC1 was chosen for further analysis.

### HP1α-HDAC1 complex regulates STAT1 expression in transcriptional level

**Fig. 5:**
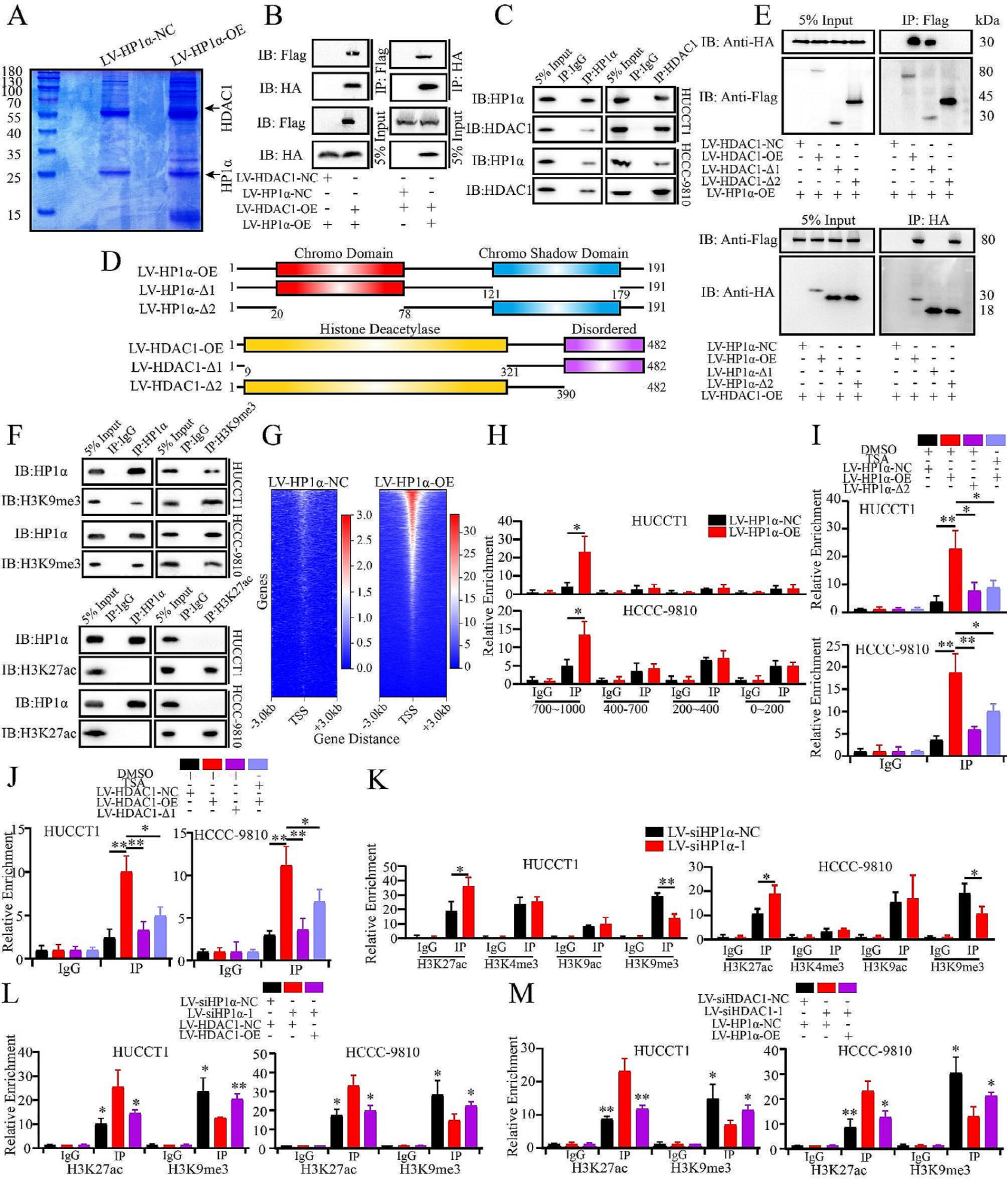
HP1α-HDAC1 complex regulates STAT1 expression in transcriptional level. **A.** The gel of the samples of control group and IP group. The sample was separated by SDS-PAGE and dyed by coomassie brilliant blue. The molecular weight of the protein ladder was shown. **B, C.** Evaluation of the interaction between HP1α and HDAC1. **B.** Exogenous co-IP. **C.** Endogenous co-IP. **D.** The scheme of the analysis for different domains of HP1α and HDAC1 protein. **E.** Mapping of the binding region of the HP1α-HDAC1 complex using exogenous co-IP. **F.** Evaluation of the interaction between HP1α and histone marks (H3K27ac and H3K9me3). **G.** Heatmap of the distribution of HP1α in the genome of HUCCT1 cells. The gradual change from red to blue represents the change in the number of peaks from most to least. **H.** Validation of the binding region of HP1α in STAT1 promoter by ChIP. **I, J.** Evaluation of the interaction between the HP1α-HDAC1 complex and the STAT1 promoter upon TSA treatment and functional domain deletion. **I.** HP1α. **J.** HDAC1. **K.** Evaluation of the distribution of histone marks in the STAT1 promoter upon HP1α knockdown using ChIP. **L, M.** Impact of HP1α-HDAC1 complex on the distribution of H3K27ac and H3K9me3. **L.** HP1α Knockdown and HDAC1 Overexpression. **M.** HDAC1 knockdown and HP1α overexpression. *P < 0.05. **P < 0.01

ESI-MS analysis was performed to validate the role of HDAC1. Among the HDACs, only HDAC1 was detected in the IP sample (Fig. 5A, S3A, Table S7), along with other proteins reported to bind to HP1α, such as CHAF1A and POGZ [35]. The interaction between HDAC1 and HP1α was validated using exogenous and endogenous co-immunoprecipitation (Fig. 5B and C). Additionally, HP1α was confirmed to bind to TRIM28 (Figure S3B), a binding protein of HDAC1 [36]. TRIM28 expression was also not regulated by HP1α (Figure S2I). The HP1α protein structure contains Chromo and Chromo Shadow domains, and the HDAC1 protein structure contains Histone Deacetylase and Disordered regions, all of which have been reported to bind to DNA-binding proteins (Fig. 5D) [3]. We found that HP1α probably binds to HDAC1 via the disordered region, whereas HDAC1 probably binds to HP1α via the chromo-shadow domain (Fig. 5E).

We failed to detect binding between HP1α and STAT1 (Figure S3C); therefore, we explored the interaction between HP1α and the STAT1 promoter region. We failed to detect any difference in luciferase activity upon HP1α overexpression, although insertion of the promoter region (1000 bp to the TSS) significantly enhanced transcription (Figure S3D). Similarly, TSA application failed to influence the transcriptional activity mediated by the STAT1 promoter, which is inconsistent with the observations in ICCA cells (Figure S3E), indicating that indirect binding to chromatin, for example, interactions via histone modifications, should be examined. Although an interaction between HP1α and H3K9me3 was observed, no binding to H3K27ac or H3K9ac was observed (Fig. 4F and S3F). To explore the distribution in local chromatin, we calculated the number of sites in each section. Most of the sites were located in the 700–1000 bp region (Figure S3G). CUT & Tag was performed to evaluate the occupancy of HP1α and histone markers (Fig. 5G, S3H). The binding sequences of the different markers are summarized in Table S8. The sequence of the HP1α binding sites was similar to that of H3K9me3, rather than acetylated histones. Most peaks in the promoters were located upstream of the 1000 bp. Except for H3K27me3, all targets exhibited peaks in the promoter region of STAT1, which was similar to the results obtained in the GEO database (Figure S3I, S3J). To extend this conclusion, we screened for all genes that contained four peaks of histone marks in the same regions (9063 genes, Figure S3K, S3L). Among these 9063 genes, 5871 contained HP1α peaks, and some genes were involved in cell cycle regulation and viral infection (Figure S3M). Among these 5871 genes, 1157 genes were significantly regulated by TSA and were highly correlated with cell cycle regulation (Figure S3N).

HP1α was found to be significantly enriched in the 700–1000 bp region of the STAT1 promoter compared to other proximal sites (Fig. 5H). The binding of HP1α to this region was inhibited by deletion of the Chromo domain or by treatment with TSA (Fig. 5I). Similarly, HDAC1 could bind to this region, and this binding was inhibited by deletion of the Histone Deacetylase domain or treatment with TSA (Fig. 5J). Additionally, only increases in H3K27ac and decreases in H3K9me3 were observed in this region after HP1α knockdown (Fig. 5K). Both H3K27ac and H3K9me3 were found to be enriched in the promoters of certain genes involved in infection and cell cycle (Figure S4A, S4B). ChIP was performed to evaluate the effect of the HP1α-HDAC1 complex on the local distribution of the two histone markers. Once either HP1α or HDAC1 was knocked down, we observed higher levels of H3K27ac and lower levels of H3K9me3, which could be partly rescued by overexpression of the other parts (Fig. 5L and M). Considering this evidence, we suggest that the HP1α-HDAC1 complex regulates the basal activation of IFN signaling by transcriptionally downregulating STAT1.

### The proliferation of ICCA cells was inhibited by directly activating IFN signaling pathway

**Fig. 6:**
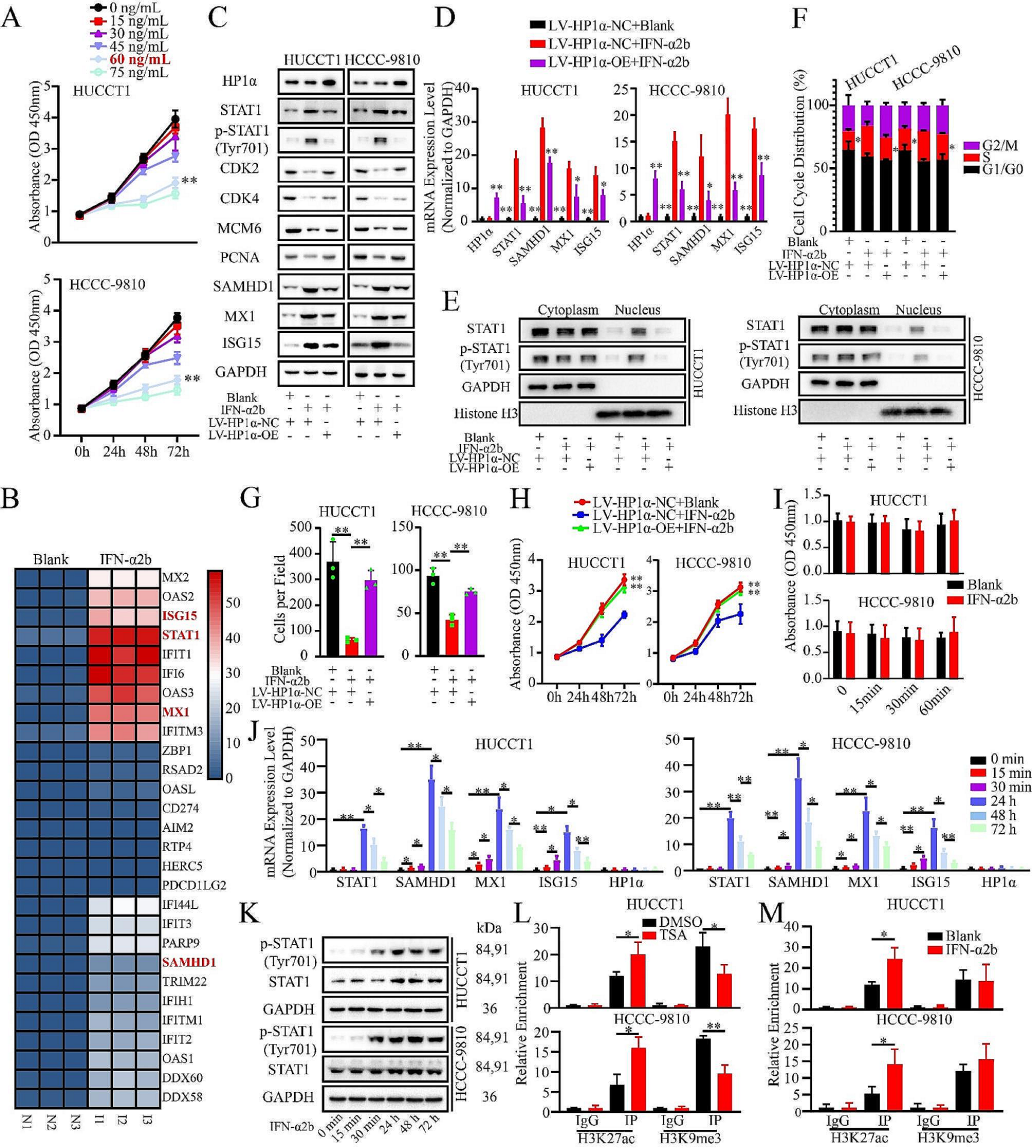
The proliferation of ICCA cells is inhibited by directly activating IFN signaling pathway. **A.** The effect of various concentrations of IFN-α2b on the proliferation of ICCA cells evaluated by CCK-8 assay. **B.** The heatmap of the RNA-seq data of IFN-α2b-treated ICCA cells. The gradual change from red to blue represents changes in gene expression from high to low. **C.** Western blot analysis of HP1α, IFN signaling participants and proliferation and cell cycle-promoting factors upon IFN-α2b treatment and HP1α overexpression. **D.** RT-qPCR analysis of the expression of HP1α, STAT1 and ISGs upon IFN-α2b treatment and HP1α overexpression. **E.** Analysis of the expression level of intranuclear STAT1 and p-STAT1 upon IFN-α2b treatment and HP1α overexpression by Western blot. **F.** The effect of IFN-α2b treatment and HP1α overexpression on the distribution of cell cycle of ICCA cells. **G-I.** The result of proliferation assay. **G.** Colony formation assay. **H.** CCK-8 assay over 24 h. **I.** CCK-8 assay within 1 h. **J.** RT-qPCR analysis of the expression of HP1α and IFN signaling participants at different point of time. **K.** Western blot analysis of the expression and the activation level of STAT1 at different point of time. **L, M.** Effect of drug treatment on the distribution of H3K9me3 and H3K27ac in the STAT1 promoter. **L.** TSA treatment. **M.** IFN-α2b treatment. *P < 0.05; **P < 0.01

To evaluate the effect of the IFN signaling pathway, IFN-α2b, a cytokine preparation that can directly and potently activate IFN signaling, was used. We attempted to determine the optimal concentration, and 60 ng/mL was selected because higher concentrations had limited inhibitory effects (Fig. 6A). HUCCT1 cells were treated and RNA-seq was performed. We found that certain ISGs were upregulated (Fig. 6B). Among the 5871 genes mentioned above, 161 were regulated by IFN-α2b, some of which were involved in IFN signaling and translation initiation (Figure S3O).

IFN-α2b significantly induced the expression of STAT1, p-STAT1, ISG15, SAMHD1, and MX1 and downregulated the protein factors (Fig. 6C and D). HP1α is not regulated by IFN-α2b. IFN-α2b treatment induced synchronous upregulation of intranuclear STAT1 and p-STAT1 (Fig. 6E). IFN-α2b inhibited proliferation and induced S-phase arrest (Fig. 6F and H, S3P). HP1α overexpression partially reversed these effects. Notably, an inhibitory effect was observed as early as 24 h after treatment (Fig. 6H). In contrast, no similar effect was observed within 1 h (Fig. 6I). To investigate this possibility, we examined the expression levels of the related molecules at different time points. Although all of these factors, except for STAT1, were upregulated before the 1 h time point, their expression was upregulated more drastically at 24 h and gradually decreased thereafter (Fig. 6J). However, HP1α expression was not affected. The protein levels of p-STAT1, but not total STAT1, increased within 1 h. Up to 24 h, the levels of both total STAT1 and p-STAT1 were elevated (Fig. 6K). IFN-α2b and TSA induced a significant increase in H3K27ac levels in the STAT1 promoter. TSA also decreased the local H3K9me3 levels (Fig. 6L and M). It could be inferred that increased total STAT1, rather than increased p-STAT1, is the underlying cause of proliferation inhibition, which is consistent with the finding that the HP1α-STAT1 axis regulates ICCA cell proliferation (Fig. 3).

### Broad-spectrum HDACi plus IFN preparation regimen improves the antiproliferation effects and inhibits the development of ICCA

**Fig. 7:**
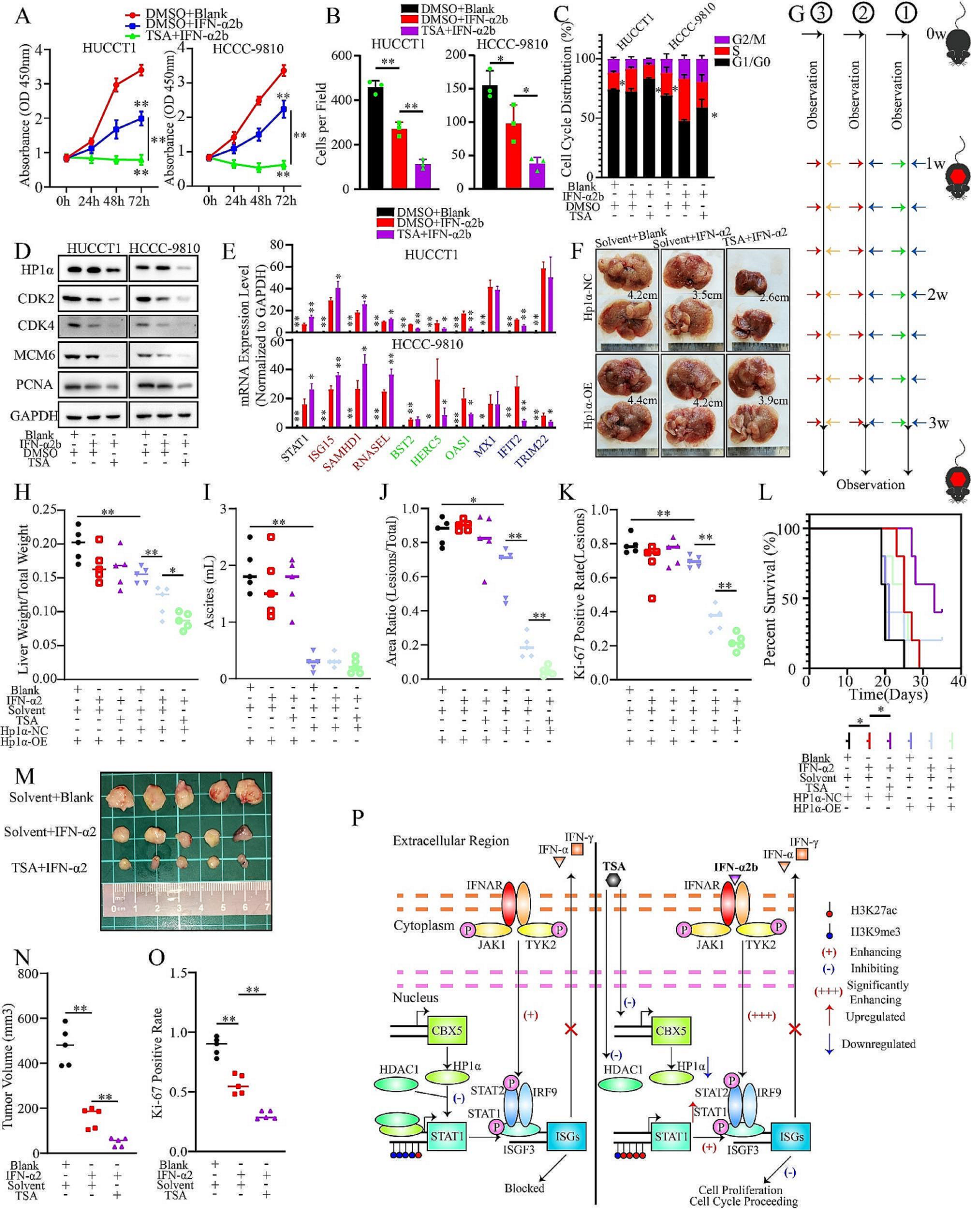
Broad-spectrum HDACi plus IFN preparation regimen maximizes the antiproliferation effects on ICCA. **A, B.** Evaluation of the proliferation inhibition induced by the TSA plus IFN-α2b regimen. **(A)** CCK-8 assay. **(B)** Colony formation assay. **Blank**, solvent for IFN-α2b, and murine IFN-α2. **C.** The distribution of cell cycle of ICCA cells. **D.** Western blot analysis of HP1α and proliferation and cell cycle-promoting factors. **E.** RT-qPCR analysis of the effect of TSA plus IFN-α2b regimen on ISG expression. **Red**, upregulated by TSA. **Blue**, downregulated by TSA. **Green**, failed to be significantly regulated by TSA. **F.** Presentation of liver lesions induced by hydrodynamic tail vein injection in the different treatment groups. **Solvent**, the recommended solvent for TSA in animal experiments. **G.** Scheme of the animal experiment. **(1)** Blank + Solvent; **(2)** IFN-α2 + Solvent; **(3)** IFN-α2 + TSA. One week after the hydrodynamic tail vein injection, different treatments were applied for two weeks. The survival status of mice was then assessed. **Blue arrow**, Solvent. **Orange arrow**, TSA. **Green arrow**, Blank. **Red arrow**, murine IFN-α2. **H-K.** Evaluation of the severity of tumorigenesis and the effect of the regimen. **H.** Ratio of liver weight to total weight. **I.** Ascites volume. **J.** ICCA lesions areas. **K.** Ki-67 positivity rate in the liver lesions. **L.** Survival analysis of the mice in different treatment groups. **M.** Presentation of the subcutaneous tumors. **N, O.** Volume **(N)** and Ki-67 positive rate of subcutaneous tumors **(O)** after different treatments. **P.** Graphic illustration of the HP1α-HDAC1-STAT1 regulatory axis in ICCA. STAT1 is directly and epigenetically repressed by the HP1α-HDAC1 complex. Treatment with TSA plus IFN significantly inhibited the proliferation of ICCA cells by targeting the HP1α-HDAC1 complex, increasing the total STAT1 level, downregulating HP1α, increasing basal IFN signaling, and directly stimulating the IFN pathway. *P < 0.05. **P < 0.01

Proliferation assays were performed to evaluate the effects of broad-spectrum HDACi and IFN preparations. Compared with IFN-α2b alone, TSA plus IFN-α2b further inhibited the proliferation of ICCA cells (Fig. 7A and B, S4C). When TSA was applied with IFN-α2b-treated cells, we observed G1/G0-phase arrest, which was similar to the recruitment effect of chemotherapy regimens (Fig. 7C). TSA plus IFN-α2b further downregulated proliferation and cell cycle factors (Fig. 7D).

Given that both reagents could induce IFN signaling, we screened for common targets using RNA-seq data of both TSA and IFN-α2b, for example, ISGs (Figure S4D, Table S9). As previously reported [37], TSA abrogated IFN-α2b-mediated upregulation of some ISGs that were either downregulated or unaffected by TSA (Fig. 7E). STAT1 and three ISGs, including SAMHD1 and ISG15, were further upregulated by TSA, which was consistent with the RNA-seq results. The upregulation of some ISGs (including MX1) was not reversed by TSA treatment (Fig. 6E). CD274 and PDCD1LG2 (encoding PD-L1 and PD-L2, respectively) were used as the positive controls as both genes were upregulated upon IFN stimulations [38]. HP1α knockdown downregulated the mRNA level of CD274 and PDCD1LG2 (Figure S4E). IFN-α2b induced the transcription of both genes, which was slightly rescued by TSA treatment (Figure S4F). The mRNA level of CD274 was positively correlated with that of HP1α in ICCA samples (Figure S4G). When HP1α was knocked down, CD274 was then downregulated (Figure S4H). IFN-α2b upregulated the expression of PD-L1, while TSA downregulated PD-L1 whether IFN-α2b was applied or not (Figure S4H).

This combination has been validated in a mouse model. We established ICCA models and found that although IFN-α2 alone ameliorated the progression of ICCA, the additional application of TSA further inhibited this oncogenic process. This therapeutic effect was abrogated by Hp1α overexpression (Fig. 7F and G, and S4I). Neither the TSA plus IFN-α2 regimen nor IFN-α2 monotherapy led to morphological changes in the kidneys (Figure S4J). All ICCA lesions were confirmed to be of an epithelial origin (Figure S4K). The TSA plus IFN-α2 regimen further reduced the weight and area of hepatic lesions and the rate of Ki-67 positivity, yet the volume of ascites was not influenced. Hp1α-overexpressing tumors were more severe and insensitive to TSA and IFN-α2 (Fig. 7H and K, S4L). Murine Cd274 was significantly upregulated upon Hp1α overexpression, which was similar to the findings in ICCA samples (Figure S4M). In addition, the TSA plus IFN-α2 regimen further improved the prognosis of the mouse model, which was not observed in the Hp1α overexpression group (Fig. 7L). Additionally, TSA plus IFN-α2 further reduced the volume of subcutaneous tumors and the rate of Ki-67 positivity (Fig. 7M and O, S4N).

In conclusion, the following mechanism is proposed. Although minimal IFN signaling is maintained, STAT1 is repressed by the HP1α-HDAC1 complex in a direct and epigenetic manner. Treatment with broad-spectrum HDACi plus IFN significantly inhibited the proliferation of ICCA cells by increasing the total STAT1 level, downregulating HP1α, increasing basal IFN signaling, and directly stimulating the IFN pathway (Fig. 7P).

## Discussion

The onset of ICCA is a global public health problem, and its incidence is increasing, particularly in regions with a high incidence of inflammatory lesions. Therefore, targeting these inflammation-related molecular mechanisms may be a therapeutic option for ICCA, although little is known about the relationship between ICCA and inflammatory signaling. In this study, we demonstrated the role of the HP1α-HDAC1-STAT1 axis as a potential target for activating the IFN signaling pathway and inhibiting ICCA cell proliferation.

As important nonhistone chromosomal protein, HP1α has been found to be associated with proper mitosis, cell cycle progression and DNA repair in multiple species and in various tissue types [3]. While heterochromatin markers are often increased in cancerous lesions [39], HP1α is upregulated in breast cancer [7]. In lung cancer, HP1α downregulation impairs cell viability by inhibiting the Wnt signaling pathway [5]. In cervical cancer, HP1α downregulation correlates with aberrant mitosis [7]. In prostate cancer, HP1α knockdown significantly induces apoptosis and growth arrest [6]. Similarly, HP1α is upregulated in ICCA. HP1α regulates proliferation, but not apoptosis or invasion, of ICCA cells. Notably, HP1α is downregulated in thyroid cancer, especially in metastatic and poorly differentiated lesions [40]. Therefore, the carcinogenic function of HP1α may be conserved in certain types of cancer.

Although the relationship between HP1α and IFN signaling remains unclear, supporting evidence has been reported. In medulloblastoma, HP1α silenced apoptosis-related inflammatory response genes. Another family member, CBX4, influenced retroviral genomic latency [41, 42]. We found that HP1α downregulation stimulated basal IFN signaling, which plays essential roles in impairing excessive proliferation via a mechanism based on ISG induction, STAT1 upregulation, and STAT1 nuclear translocation [10]. Additionally, we demonstrated that STAT1 is the central node in the crosstalk between HP1α and IFN signaling, based on the following observations. First, HP1α failed to regulate autocrine IFN and related signaling pathways. Second, the function of HP1α was unidirectionally regulated by the activated upstream kinase and blocked by IRF9 knockdown and downstream ISGF3 elimination. Third, intranuclear STAT1 and p-STAT1 were both synchronously upregulated upon HP1α knockdown. Fourth, HP1α expression remained stable upon the application of the treatments described above. The HP1α-STAT1 axis was further validated to inhibit the proliferation of ICCA cells in vivo and in vitro via the induction of IFN signaling and upregulation of ISGs, which seemed to conflict with the consequence of stemness maintenance arising from the passive regulation of STAT1 and IFN signaling in breast cancer [43, 44].

Next, we demonstrated that STAT1 might be regulated by histone acetylation using online database analysis, TSA treatment assays, and RNA-seq analysis. This conclusion was further validated in studies using cell lines derived from other tissues. Rampazzo et al. noted that TSA inhibited the IFN pathway in glioblastoma cells [45]. We observed the downregulation of some ISGs in the IFN pathway, which could be partially explained by differences among tissues [34]. However, we hypothesized that IFN signaling was activated in ICCA cells, despite its limited level, because TSA upregulated another group of critical genes, STAT1 and STAT2. In line with this conclusion, we found that STAT1 was regulated by HDAC1, but not by HDAC2 or HDAC6, by siRNA transfection or specific inhibitor treatment. HDACs can catalyze the removal of acetyl groups from lysine residues in the amino-terminal region of histone H3 and sometimes function as oncogenes [34, 37, 44]. In view of the common functions of DNA binding and increasing chromosome density, the interaction between HP1α and HDAC1 in the five HDACs in the database was verified in ICCA cells using co-IP and ESI-MS. This interaction was reported casually by Hauri et al., Li et al., and Zhang et al., but the binding pattern has not been discussed in detail [46–48]. Although crosstalk between HDACs and STAT1 has been reported previously, most studies have focused on the activation status rather than the transcriptional level of STAT1 [49, 50]. The HP1α-HDAC1 complex was located in the STAT1 promoter region in a H3K9me3-dependent manner, and this location was validated using CUT&Tag, co-IP, and ChIP. Compared to functional domain deletion, TSA application only partially abrogated this interaction in the local environment and increased the basal intensity of IFN signaling.

Unlike basal activation by TSA, direct activation of the IFN pathway by IFN-α2b drastically induced STAT1 and ISG expression in a time-dependent manner. Both activation mechanisms significantly inhibited the proliferation of ICCA cells, which was rescued by HP1α overexpression, indicating a role for HP1α in TSA and IFN-α2b resistance. This phenomenon may be explained by transcriptional regulation. We found that many genes, including STAT1, contained multiple histone marks and were occupied by HP1α. Some of these genes are involved in antiproliferative and antimicrobial processes. These are critical factors in the IFN pathway. TSA-regulated genes influence cell proliferation. IFN-α2b-regulated genes tend to influence antimicrobial function. Notably, we identified many terms related to protein translation initiation that were associated with the regulation of protein expression. This might explain why no proliferation-related terms were enriched in the RNA-seq data, even though the protein factors were significantly differentially regulated. Hansen et al. demonstrated that methylated H3K9 can impair IFN signaling activation in acute myeloid leukemia [51]. We found that HP1α knockdown affected the local distribution of histone modifications; for example, more H3K27ac and less H3K9me3, although the overall level of H3K27ac was stable. TSA exerted similar effects, whereas IFN-α2b increased only H3K27ac levels. TSA significantly downregulated HP1α expression, suggesting a potential strategy for the treatment of HP1α-positive ICCA. Broad-spectrum HDACi could not be replaced by specific HDACi for the limited anti-proliferative effects.

In this study, we evaluated the treatment effect of a broad-spectrum HDACi plus IFN preparation regimen and found that this combination maximized the antiproliferative effect. This regimen has been shown to be promising in two distinct murine models. This combination did not result in a visible injury to the kidneys. HP1α overexpression enhances resistance to this regimen. Another consequence of this regimen is regulation of IFN-related genes. Both PD-L1 and PD-L2 are upregulated upon IFN treatment, which could be regarded as a side effect of IFN preparations [10]. TSA treatment slightly reversed this effect. Similarly, the combination of IFN-α2b and TSA downregulated some ISGs, particularly those that were not elevated by TSA monotherapy, as previously reported in acute myeloid leukemia [37]. However, we believe that this contradiction can be partially explained by the high-throughput assessment. TSA treatment also increased the expression of some ISGs (represented by SAMHD1). TSA occasionally failed to overcome IFN-α2b stimulation (represented by MX1). Therefore, TSA seemed to have little effect on the antitumor ISG profile of IFN-α2b based on the finding that proliferation was significantly impaired by this combination, which supports its application.

Our study has several advantages. First, we detected only the HP1α-HDAC1 complex in ICCA cells, although other HDACs can also bind to other tumors. Second, we elucidated the relationship between HP1α and antitumor IFN signaling. STAT1 was identified as the central node and is regulated by the HP1α-HDAC1 complex. Third, to better utilize this molecular mechanism (a common biological function, a common function in regulating histone marks, and the combination of basal and direct activation), we validated the effect of the IFN-α2b plus TSA regimen. However, this method also has several disadvantages. First, this study was limited by the relatively low incidence of ICCA; therefore, multicenter ICCA cohorts were not obtained. Second, the detailed molecular mechanism underlying the regulation of each ISG, especially MX1, remains unclear, although the induction of additional ISGs could be interpreted by the molecular mechanism we found. Third, although the regimen in this study was proven to be promising, it was not validated in ICCA patients, which requires future work and more evidence.

## Conclusions

HP1α-HDAC1 complex influences interferon pathway activation by directly and epigenetically regulating STAT1 in transcriptional level. The broad-spectrum HDACi plus interferon preparation regimen inhibits ICCA development, providing feasible strategies for ICCA treatment. Targeting the HP1α-HDAC1-STAT1 axis is a possible strategy for treating ICCA, especially HP1α-positive cases, which requires more evidence and clinical trials.

### Electronic supplementary material

Below is the link to the electronic supplementary material.

Supplementary Material 1

Supplementary Material 2

Supplementary Material 3

Supplementary Material 4

Supplementary Material 5

Supplementary Material 6

Supplementary Material 7

Supplementary Material 8

Supplementary Material 9

Supplementary Material 10

Supplementary Material 11

Supplementary Material 12

Supplementary Material 13

## Data Availability

The datasets downloaded, generated, and analyzed during the present study are available from the corresponding author upon reasonable request. Raw RNA-seq and CUT & Tag data were uploaded to the GEO database (GSE252094, GSE252095, GSE252096, and GSE252098).

## Abbreviations

CCA: Cholangiocarcinoma
ICCA: Intrahepatic cholangiocarcinoma
HCCA: Hilar cholangiocarcinoma
ECCA: Extrahepatic cholangiocarcinoma
GBC: Gallbladder cancer
HP1α: Heterochromatin Protein 1α
H3K9me3: Trimethylated H3K9
IFN: Interferon
PARP: Poly (ADP-ribose) polymerase
IFN-I, II, III: Type I, II and III interferon signaling
FDA: The Food and Drug Administration
STAT1: Signal Transducer and Activator of Transcription 1
HDACi: Histone deacetylase inhibitor
SPF: Specific pathogen-free
MX1: MX dynamin-like GTPase 1
ISG: IFN-stimulated gene
SAMHD1: SAM domain and HD domain 1
JAK1: Janus kinase 1
TYK2: Tyrosine kinase 2
TIC: Transcription initiation complex
ISGF3: IFN‐stimulated Gene Factor 3
H3K27ac: Acetylated H3K27
HDAC: Histone deacetylase
CHAF1A: Chromatin Assembly Factor 1 Subunit A
POGZ: Pogo Transposable Element with ZNF Domain
TRIM28: Tripartite Motif Containing 28
p-STAT1: Phosphorylated STAT1
