# Correction: Unveiling the role of HP1α-HDAC1-STAT1 axis as a therapeutic target for HP1α-positive intrahepatic cholangiocarcinoma

**DOI:** 10.1186/s13046-024-03112-w

**Published:** 2024-07-05

**Authors:** Fei Xiong, Da Wang, Wei Xiong, Xin Wang, Wen-hua Huang, Guan-hua Wu, Wen-zheng Liu, Qi Wang, Jun-sheng Chen, Yi-yang Kuai, Bing Wang, Yong-jun Chen

**Affiliations:** 1 Department of Biliary-Pancreatic Surgery, Tongji Hospital, Tongji Medical College, Huazhong University of Science and Technology, No. 1095 Jiefang Road, Wuhan, Hubei 430074 China; 2 Department of General Surgery, Beijing Friendship Hospital, Capital Medical University Beijing, Beijing, 100050 China; 3 Department of Orthopedics, Tongji Hospital, Tongji Medical College, Huazhong University of Science and Technology, Wuhan, 430074 Hubei China; 4 Departement of Pediatric Surgery, Wuhan Children’s Hospital, Tongji Medical College, Huazhong University of Science and Technology, Hubei, 430016 China; 5 Department of Emergency, Tongji Hospital, Tongji Medical College, Huazhong University of Science and Technology, Wuhan, Hubei 430074 China

**Correction: J Exp Clin Cancer Res 43, 152 (2024)**

10.1186/s13046-024-03070-3.

Following publication of the original article [1], the authors would like to add another affiliation to Fei Xiong. Tongji Hospital where he graduated has to be added to his affiliation details. The revised affiliations are shown below:

Fei Xiong**1**,2†, Da Wang1†, Wei Xiong3†, Xin Wang4, Wen-hua Huang5, Guan-hua Wu1, Wen-zheng Liu1, Qi Wang1, Jun-sheng Chen1, Yi-yang Kuai1, Bing Wang1* and Yong-jun Chen1*.

The correction does not affect the overall result or conclusion of the article. The original article has been corrected.
