# Supplementary material for: Unveiling the role of HP1α-HDAC1-STAT1 axis as a therapeutic target for HP1α-positive intrahepatic cholangiocarcinoma
Source: J Exp Clin Cancer Res. 2024 May 30;43:152. doi: 10.1186/s13046-024-03070-3 (PMC11137995; doi:10.1186/s13046-024-03070-3)
Supplement: Supplementary file 8 — Supplementary Material 8 [file 13046_2024_3070_MOESM8_ESM.docx]

Table S8. The binding sequence of HP1α and different histone marks.

| Number | Motif (H3K27ac) | Sites | Width | E-value |
| --- | --- | --- | --- | --- |
| 1 | 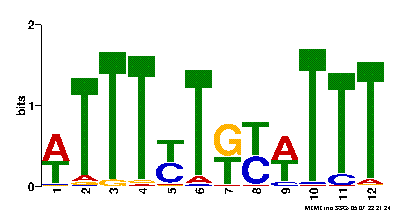 | 412 | 12 | 1.2e-323 |
| 2 | 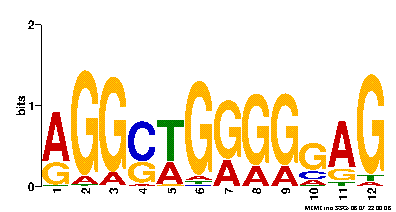 | 599 | 12 | 7.8e-256 |
| 3 | 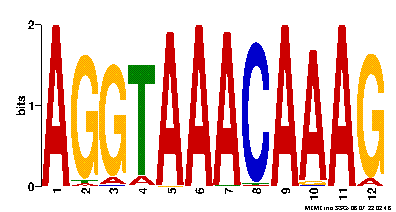 | 109 | 12 | 3.2e-205 |
| 4 | 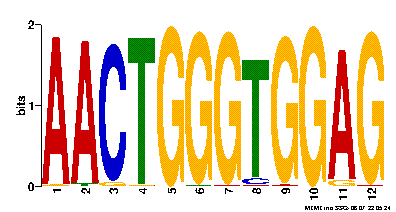 | 111 | 12 | 6.4e-208 |
| 5 | 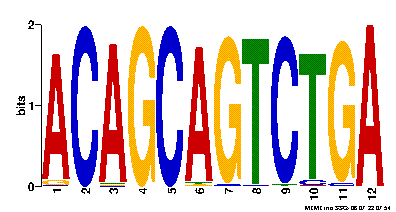 | 107 | 12 | 2.3e-201 |
| 6 | 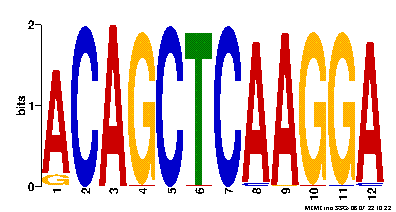 | 98 | 12 | 2.7e-191 |

| Number | Motif (H3K27me3) | Sites | Width | E-value |
| --- | --- | --- | --- | --- |
| 1 | 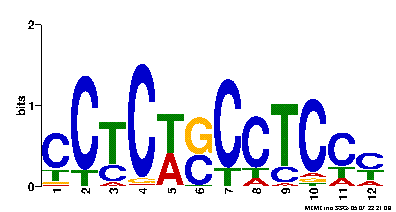 | 576 | 12 | 2.2e-61 |
| 2 | 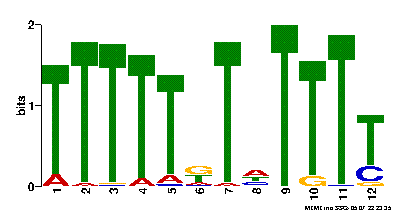 | 133 | 12 | 3.7e-54 |
| 3 | 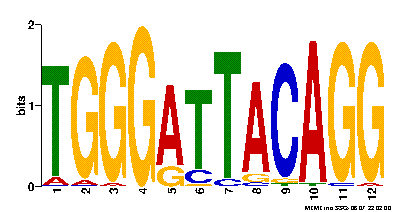 | 67 | 12 | 5.9e-37 |
| 4 | 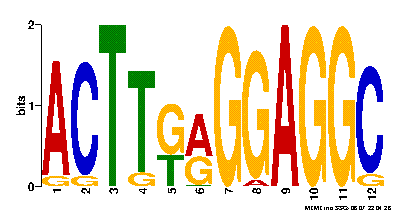 | 55 | 12 | 3.3e-25 |
| 5 | 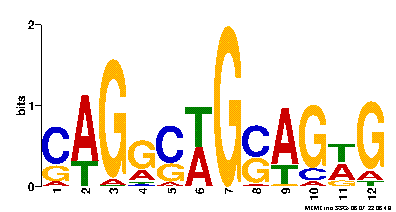 | 489 | 12 | 2.6e-17 |
| 6 | 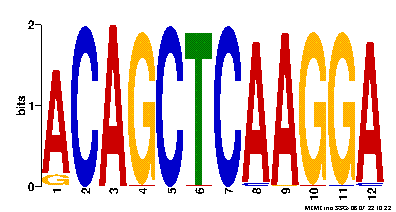 | 38 | 12 | 3.0e-3 |

| Number | Motif (H3K4me3) | Sites | Width | E-value |
| --- | --- | --- | --- | --- |
| 1 | 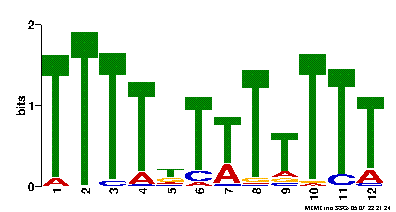 | 343 | 12 | 1.1e-185 |
| 2 | 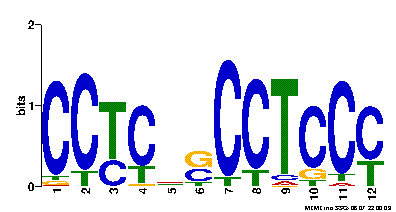 | 513 | 12 | 7.7e-155 |
| 3 | 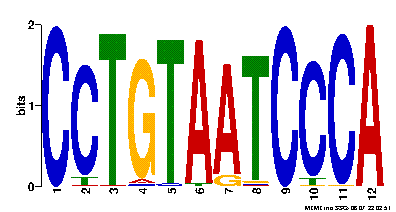 | 79 | 12 | 1.1e-103 |
| 4 | 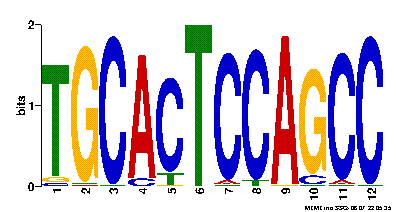 | 60 | 12 | 3.5e-51 |
| 5 | 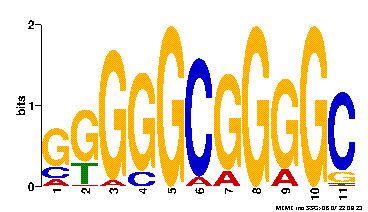 | 139 | 11 | 9.4e-44 |
| 6 | 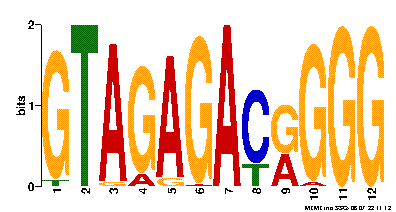 | 55 | 12 | 2.5e-42 |

| Number | Motif (H3K9ac) | Sites | Width | E-value |
| --- | --- | --- | --- | --- |
| 1 | 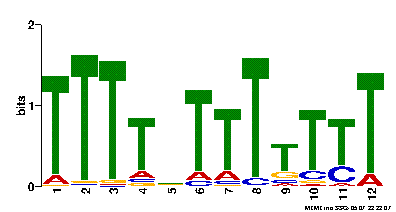 | 495 | 12 | 7.8e-362 |
| 2 | 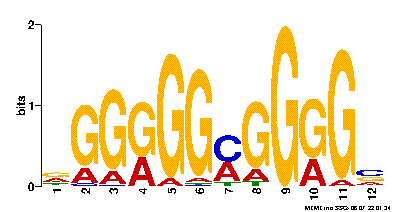 | 594 | 12 | 5.5e-165 |
| 3 | 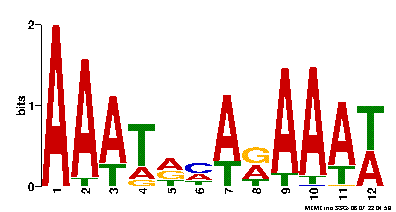 | 320 | 12 | 7.5e-99 |
| 4 | 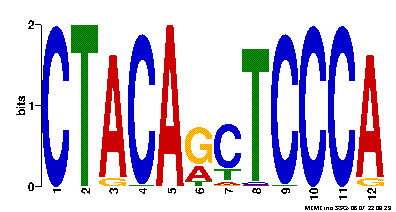 | 73 | 12 | 3.6e-68 |
| 5 | 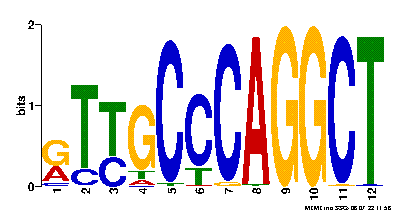 | 111 | 12 | 2.3e-57 |
| 6 | 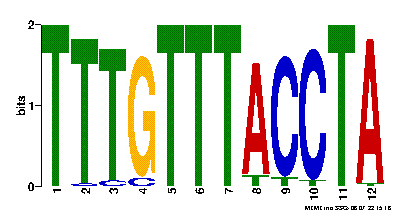 | 41 | 12 | 3.8e-53 |

| Number | Motif (H3K9me3) | Sites | Width | E-value |
| --- | --- | --- | --- | --- |
| 1 | 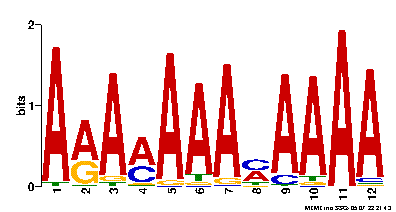 | 390 | 12 | 4.8e-498 |
| 2 | 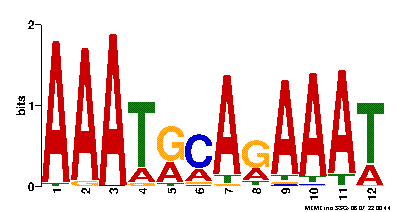 | 338 | 12 | 5.5e-321 |
| 3 | 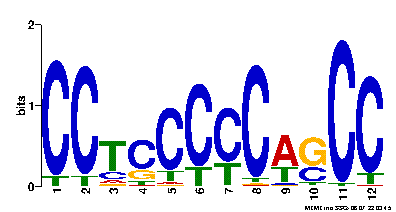 | 600 | 12 | 4.2e-275 |
| 4 | 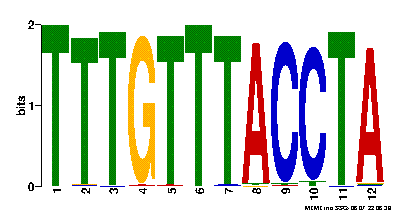 | 113 | 12 | 1.7e-259 |
| 5 | 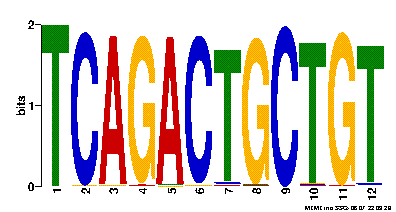 | 117 | 12 | 2.8e-238 |
| 6 | 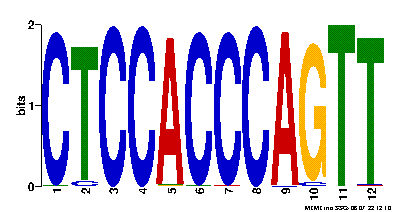 | 114 | 12 | 3.1e-237 |

| Number | Motif (HP1α) | Sites | Width | E-value |
| --- | --- | --- | --- | --- |
| 1 | 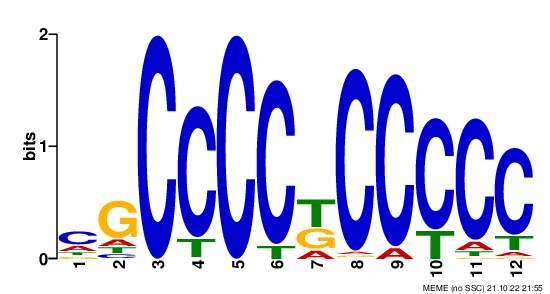 | 275 | 12 | 6.0e-148 |
| 2 | 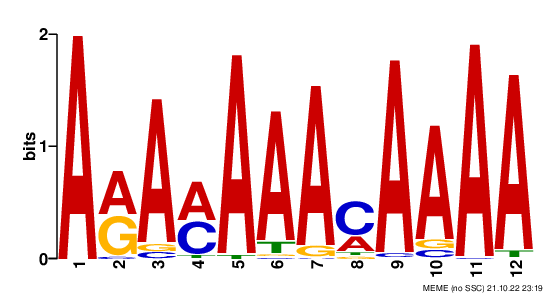 | 188 | 12 | 1.3e-137 |
| 3 | 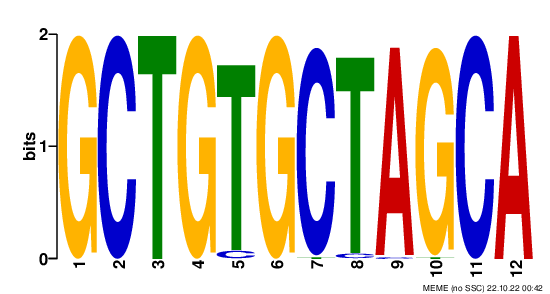 | 67 | 12 | 3.1e-126 |
| 4 | 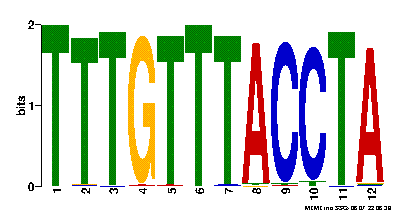 | 67 | 12 | 7.8e-126 |
| 5 | 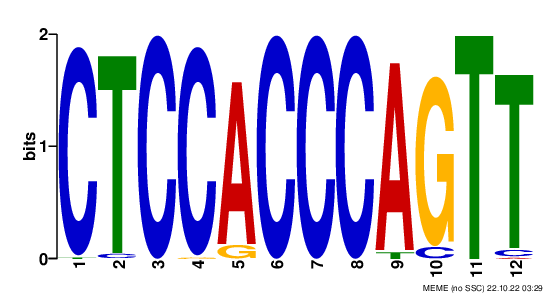 | 72 | 12 | 1.9e-119 |
| 6 | 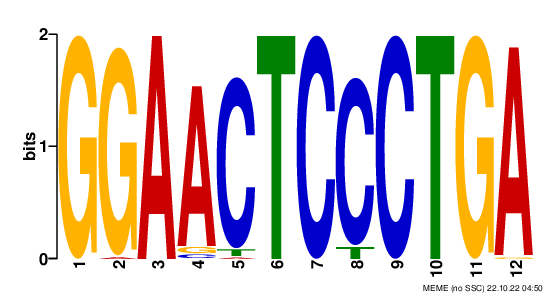 | 68 | 12 | 3.1e-237 |
