# Supplementary figures and images for: Unveiling the role of HP1α-HDAC1-STAT1 axis as a therapeutic target for HP1α-positive intrahepatic cholangiocarcinoma
Source: J Exp Clin Cancer Res. 2024 May 30;43:152. doi: 10.1186/s13046-024-03070-3 (PMC11137995; doi:10.1186/s13046-024-03070-3)

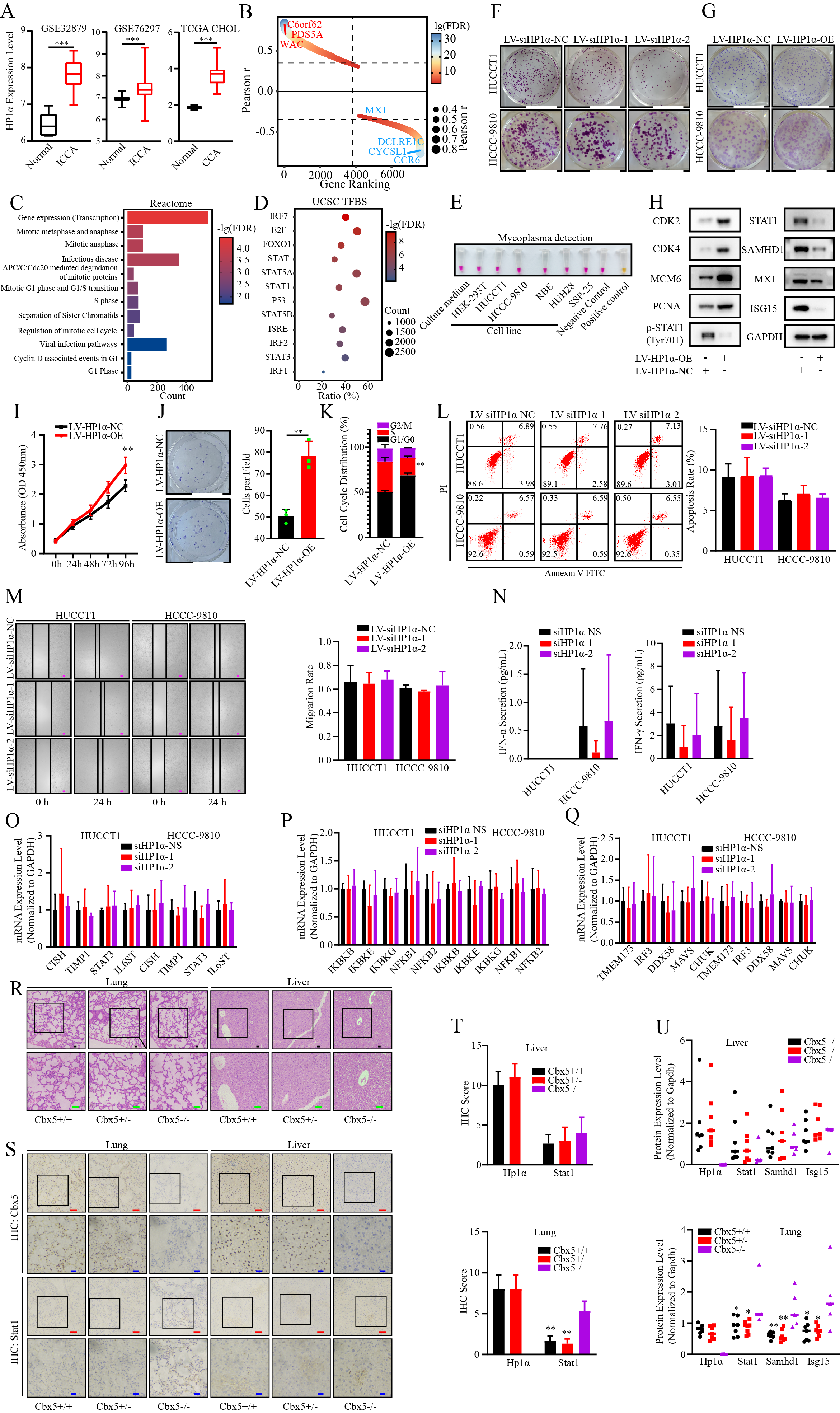

Supplement: Supplementary file 10 — Supplementary Material 10 [file 13046_2024_3070_MOESM10_ESM.tif]

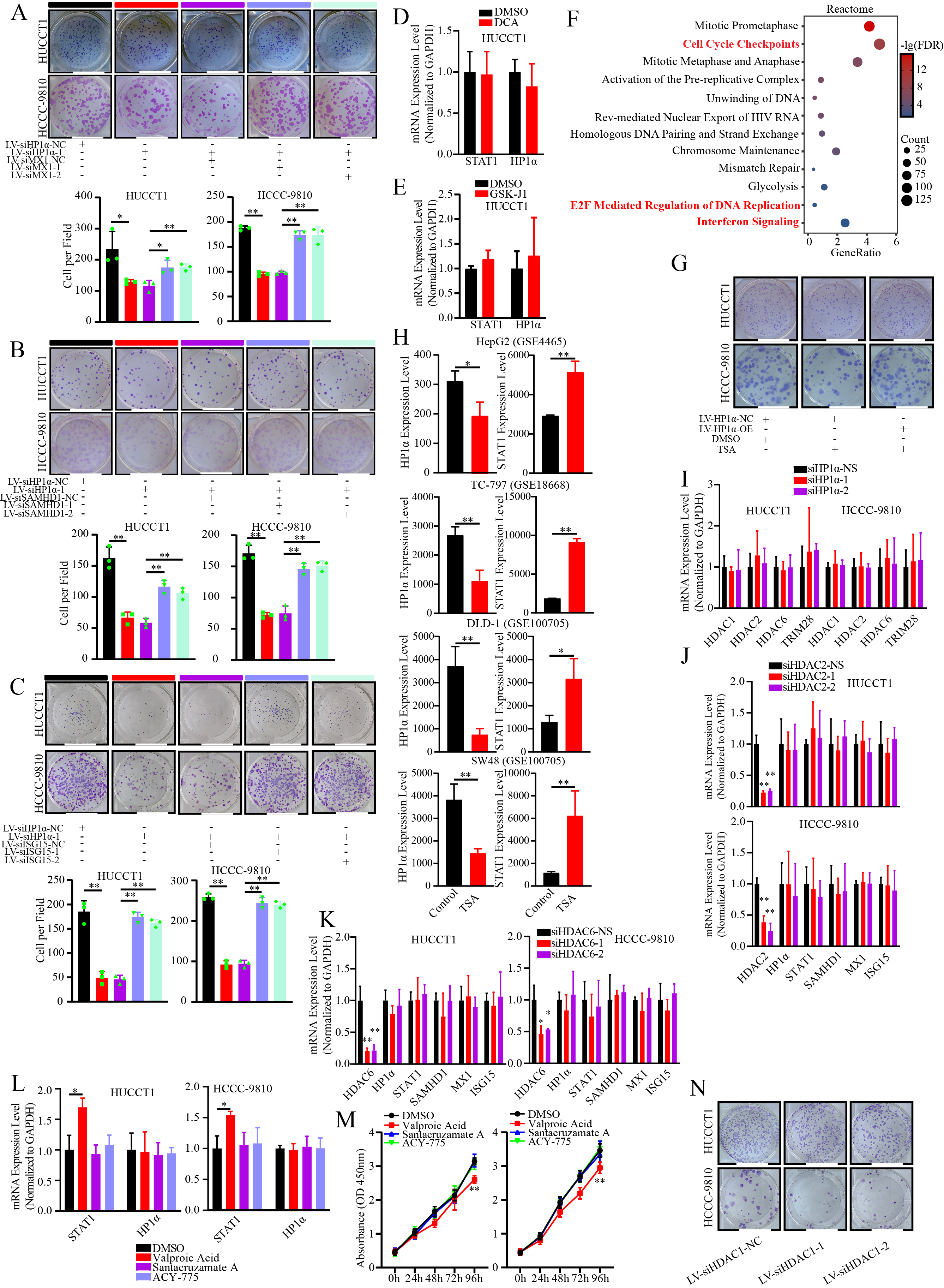

Supplement: Supplementary file 11 — Supplementary Material 11 [file 13046_2024_3070_MOESM11_ESM.tif]

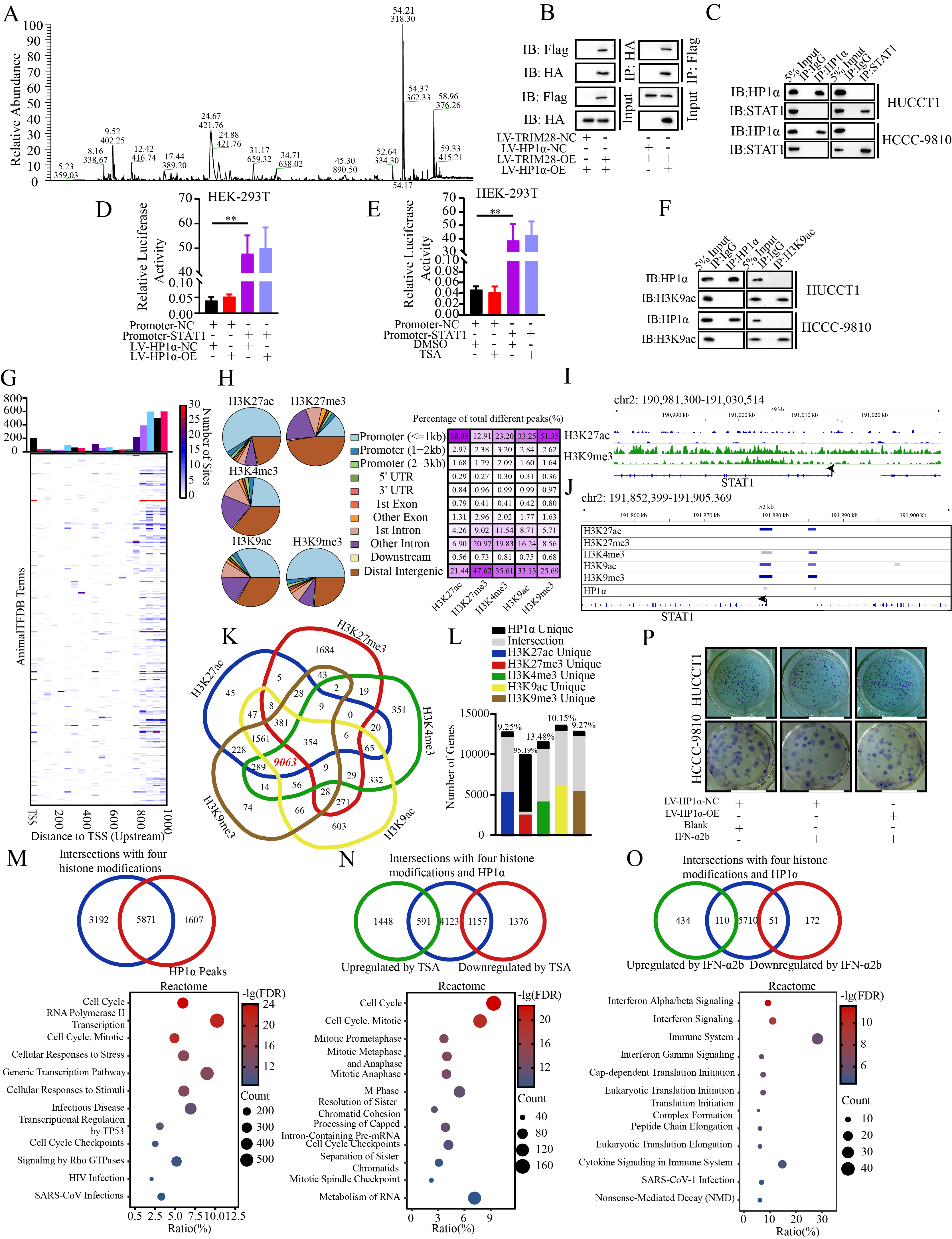

Supplement: Supplementary file 12 — Supplementary Material 12 [file 13046_2024_3070_MOESM12_ESM.tif]

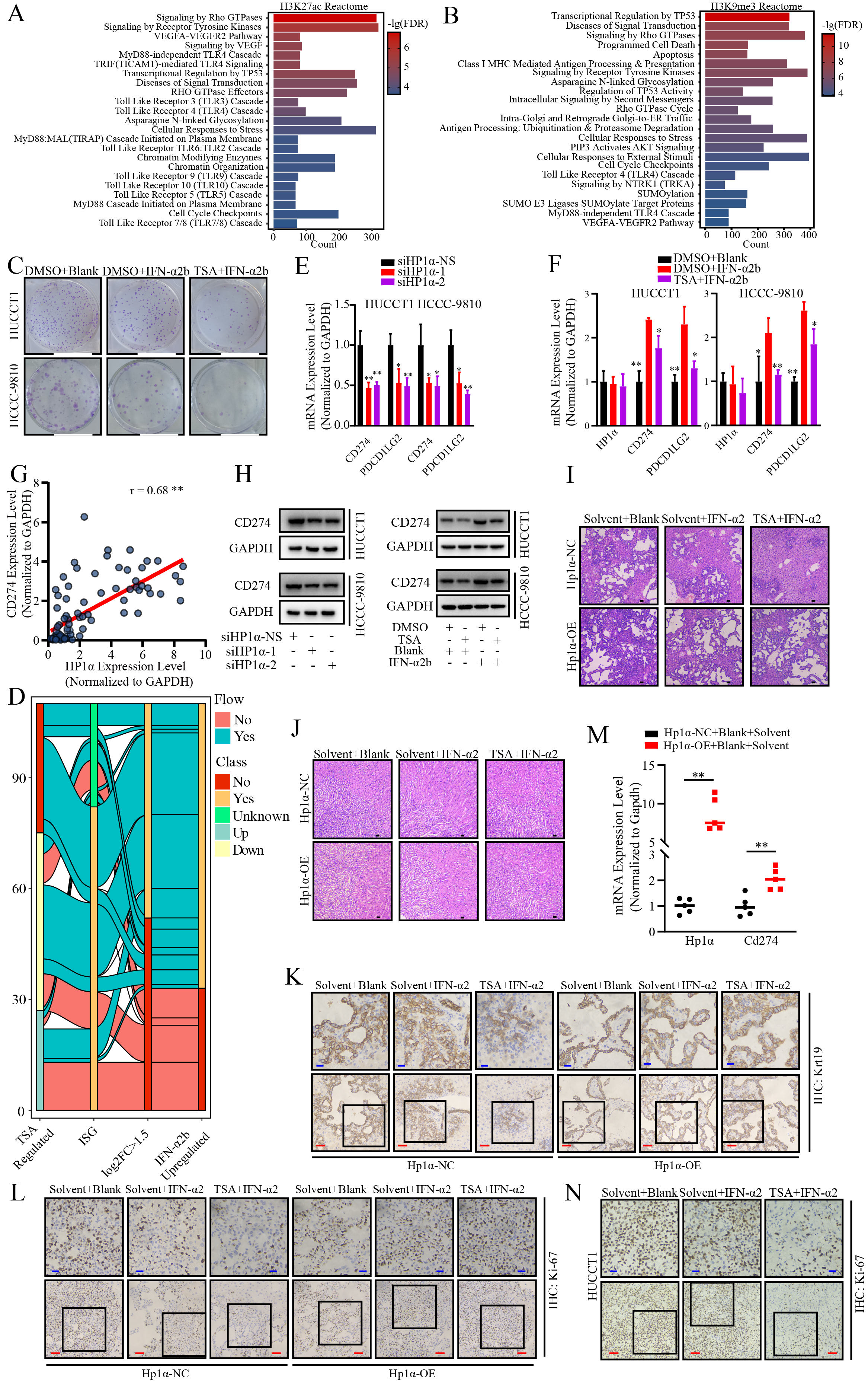

Supplement: Supplementary file 13 — Supplementary Material 13 [file 13046_2024_3070_MOESM13_ESM.tif]
